# CuZnAl-Oxide Nanopyramidal Mesoporous Materials for the Electrocatalytic CO_2_ Reduction to Syngas: Tuning of H_2_/CO Ratio

**DOI:** 10.3390/nano11113052

**Published:** 2021-11-13

**Authors:** Hilmar Guzmán, Daniela Roldán, Adriano Sacco, Micaela Castellino, Marco Fontana, Nunzio Russo, Simelys Hernández

**Affiliations:** 1 CREST Group, Department of Applied Science and Technology (DISAT), Politecnico di Torino, C.so Duca Degli Abruzzi, 24, 10129 Turin, Italy; hilmar.guzman@polito.it (H.G.); daniela.roldan@polito.it (D.R.); micaela.castellino@polito.it (M.C.); nunzio.russo@polito.it (N.R.); 2 IIT—Istituto Italiano di Tecnologia, Via Livorno, 60, 10144 Turin, Italy; adriano.sacco@iit.it (A.S.); marco.fontana@iit.it (M.F.)

**Keywords:** CO_2_ electrochemical reduction, CO_2_ utilization, metal oxide electrocatalysts, electrochemical impedance spectroscopy

## Abstract

Inspired by the knowledge of the thermocatalytic CO_2_ reduction process, novel nanocrystalline CuZnAl-oxide based catalysts with pyramidal mesoporous structures are here proposed for the CO_2_ electrochemical reduction under ambient conditions. The XPS analyses revealed that the co-presence of ZnO and Al_2_O_3_ into the Cu-based catalyst stabilize the CuO crystalline structure and introduce basic sites on the ternary as-synthesized catalyst. In contrast, the as-prepared CuZn- and Cu-based materials contain a higher amount of superficial Cu^0^ and Cu^1+^ species. The CuZnAl-catalyst exhibited enhanced catalytic performance for the CO and H_2_ production, reaching a Faradaic efficiency (FE) towards syngas of almost 95% at −0.89 V vs. RHE and a remarkable current density of up to 90 mA cm^−2^ for the CO_2_ reduction at −2.4 V vs. RHE. The physico-chemical characterizations confirmed that the pyramidal mesoporous structure of this material, which is constituted by a high pore volume and small CuO crystals, plays a fundamental role in its low diffusional mass-transfer resistance. The CO-productivity on the CuZnAl-catalyst increased at more negative applied potentials, leading to the production of syngas with a tunable H_2_/CO ratio (from 2 to 7), depending on the applied potential. These results pave the way to substitute state-of-the-art noble metals (e.g., Ag, Au) with this abundant and cost-effective catalyst to produce syngas. Moreover, the post-reaction analyses demonstrated the stabilization of Cu_2_O species, avoiding its complete reduction to Cu^0^ under the CO_2_ electroreduction conditions.

## 1. Introduction

CO_2_ is an essential trace gas in the atmosphere of the Earth. It plays a vital role as a necessary ingredient in the life cycle of plants and animals. CO_2_ is present in numerous organic compounds, in the air we breathe, in oil and natural gas deposits, in volcanoes, as well as in the sea. Although it is a normal, if minor, constituent of air, high concentrations of CO_2_ in the atmosphere can be dangerous for the environment and living species of the Earth, according to the Intergovernmental Panel report on Climate Change (IPCC) [1]. While carbon dioxide is part of the carbon cycle that allows life to be sustainable on the planet and is naturally present in the atmosphere, this gas and others, such as methane, nitrogen oxide, and ozone, are greenhouse gases (GHG). The increase in the levels of these gases (as a product of human industrial activities) contributes to the formation of a gaseous layer in the atmosphere that hinders heat radiation, thus increasing the Earth’s temperature and contributing to climate change [2]. In the case of CO_2_—the key contributor to global climate change in the atmosphere—its atmospheric level is increasing more than ever in the history of the Earth: It reached 410 ppm in June 2019 and will probably reach about 600 ppm by 2100, if the CO_2_ emission continues to follow the current trend [3]. Even though anthropogenic emissions may stop, the world will not be close to the stabilization of GHG concentrations, therefore compromising the Earth with several centuries of rising world temperatures. There is an urgent need to mitigate the consequences of climate change. Therefore, it is important to explore the different alternatives for the management of GHGs and, in this particular case, CO_2_ [4]. The simultaneous need to reduce GHG emissions and increase our energy supply makes the electrochemical reduction of CO_2_ (ER-CO_2_) a very attractive alternative. Within this context, this research seeks to demonstrate that ER-CO_2_ is an effective method to transform CO_2_ into added-value products, e.g., syngas (an H_2_/CO mixture) [3,5,6]. Nonetheless, the free Gibbs energy of CO_2_ is relatively high (ΔG^0^ = −400 kJ mol^−1^). Consequently, a substantial energy input, optimized reaction conditions, and catalysts with high activity and stability are needed to convert it [7].

The ER-CO_2_ is particularly attractive due to its compatibility with renewable energy systems, low environmental impact, low operative costs, high flexibility, and capability to store CO_2_ into high energy density products [8,9]. Indeed, this kind of system can be coupled with a renewable electricity source, such as solar or wind energy, and use water for the in-situ generation of protons (H^+^), under mild reaction conditions, to produce carbon-neutral fuels or industrial chemicals that are conventionally produced from fossil fuels [5,6,10]. This approach is especially relevant considering that the global energy demand for 2050 will double the current one (from 12 to 30 terawatts) and will be more than triple by the end of the century. This increased energy demand will create strong competition due to the inevitable depletion of fossil fuel reserves, which would affect the current quality of life in many countries that depends on the low cost of energy supply [11].

CO is one of the only sustainable products with a net present value (NPV) of USD 13.5 million from a current techno-economic evaluation [12]. In fact, CO production follows the simplest electrocatalytic CO_2_ reduction reaction with 2 e^−^ transfer (see Equation (2)). For this reason, it is the most studied product in the literature until now, which presents the best-obtained performances. Within this context, noble metals such as silver (Ag) [13] and gold (Au) [14] are the most used electrocatalysts for CO (or syngas: H_2_/CO) production [6]. Indeed, Ag-based catalysts have achieved Faradaic efficiencies > 70% and current densities > 50 mA cm^−2^ [15,16]. On the contrary, copper-based electrodes with different structures (copper nanoparticles, oxide-derived copper, and copper composites) show increased yields for hydrocarbon and oxygenate products, while their selectivity towards CO is usually low [10,17,18,19]. Nevertheless, it has been demonstrated that by modifying the structure and surface composition of the Cu-based materials, it is possible to tune the selectivity of the reaction pathway towards different H_2_/CO ratios [20]. This is a good compromise since Cu catalysts are cheaper and more feasible than Au and Ag metal catalysts. Recently, Zn has also gained attention since it is highly abundant and considerably cheaper than Cu. Indeed, Zn has a weaker CO adsorption strength than Au [21,22]. D. Meyer and D. Marx [23] suggested that ZnO is a better electrocatalyst than Zn for the CO formation due to its CO adsorption strength on the surface of the catalyst. Therefore, combining the ability of Cu for strongly binding protons [24] to ZnO could control the hydrogen evolution reaction (HER) and the ER-CO_2_. The combination of Cu and ZnO supported on a metal oxide such as Al_2_O_3_ has been widely used for thermocatalytic methanol synthesis from syngas mixtures at high temperatures and pressures at an industrial scale [25]. Nevertheless, Thomas F. Jaramillo et al. [26] prepared a thin film of Cu supported on Al_2_O_3_ in order to promote the epitaxial growth in <111> orientations for the electroreduction of CO_2_ to fuels and chemicals. The results revealed that this material was more selective for H_2_ and CO formation than for >2e^−^ oxygenates products.

The conversion of CO_2_ is a complex multistep reaction involving shared intermediates and multiple reaction pathways. Moreover, the standard reduction potentials for the ER CO_2_ (at pH = 0) are within 100–200 mV from the equilibrium potential for the hydrogen evolution reaction. The formation of the different products in the cathodic side can be explained according to Reactions (1)–(10):
Cathode:E° (V vs. NHE, pH = 0)
2H^+^ + 2 e^−^ ⇄ H_2_0.000(1)CO_2_ + 2H^+^ + 2 e^−^ ⇄ CO + H_2_O−0.105(2)CO_2_ + 2H^+^ + 2 e^−^ ⇄ HCOOH−0.169(3)CO_2_ + 6H^+^ + 6 e^−^ ⇄ CH_3_OH + 6H_2_O−0.017(4)CO_2_ + 8H^+^ + 8 e^−^ ⇄ CH_4_ + 2H_2_O0.169(5)2CO_2_ + 10H^+^ + 10 e^−^ ⇄ CH_3_CHO + 3H_2_O0.050(6)2CO_2_ + 12H^+^ + 12 e^−^ ⇄ C_2_H_5_OH + 3H_2_O0.084(7)3CO_2_ + 16H^+^ + 16 e^−^ ⇄ CH_3_(CO)CH_3_ + 5H_2_O−0.140(8)3CO_2_ + 16H^+^ + 16 e^−^ ⇄ CH_3_CH_2_CHO + 5H_2_O0.140(9)3CO_2_ + 18H^+^ + 18 e^−^ ⇄ C_3_H_7_OH + 5H_2_O0.099(10)


Despite the advantages of the ER-CO_2_ system [8,9], further efforts are needed to develop electrocatalysts with high selectivity (i.e., low by-products formation) and optimize the electrochemical process conditions, as well as the cell design in order to decrease mass transfer limitation issues and pursue high production rates (high current densities). 

In this study, copper oxide (CuO), zinc oxide (ZnO), and aluminium oxide (Al_2_O_3_) catalyst materials with different ratios of three oxides were synthesized by the co-precipitation method in order to evaluate their catalytic performance for the electrochemical conversion of CO_2_ to the added-value products. Ternary CuZnAl-based catalysts are traditionally used for the CO_2_ hydrogenation to CO and methanol at high temperature and pressure [25]. However, we have recently demonstrated that a commercial catalyst with this composition, although constituted by insulating components, can be used for the ER-CO_2_ [27]. In this work, a simple and scalable co-precipitation method was employed to develop a CuZnAl-oxide based catalyst with a novel structure constituted by a mesoporous corsage of nanopyramids that grew from a common nucleation point. The relationships between the composition, structure, and electrochemical activity of different CuZnAl-, CuZn-, and Cu-catalysts were studied through different characterization methods (such as N_2_ adsorption isotherms, X-ray diffraction, X-ray photoelectron spectroscopy, field-emission scanning electron microscopy, and transmission-electron microscopy) and electrochemical techniques (voltammetry, amperometry, and electrochemical impedance spectroscopy), to explain the improved performance and selectivity of the CuZnAl catalysts for the electrocatalytic CO_2_ conversion to syngas. Our results revealed that the co-addition of ZnO and Al_2_O_3_ promotes a higher porosity and smaller size of CuO nanocrystals than in the case of the CuZn- and Cu-based catalysts, which contributed to the control of the CO formation over H_2_ to produce tunable syngas mixtures in the function of the applied reduction potential.

## 2. Materials and Methods

### 2.1. Materials

Copper (II) nitrate trihydrate (CuN_2_O_6_·3H_2_O, 99–104%). Zinc nitrate hexahydrate crystallized (Zn(NO_3_)_2_·6H_2_O, ≥99.0%). Aluminium nitrate nonahydrate (AlN_3_O_9_·9H_2_O, ≥98%). Potassium bicarbonate (KHCO_3_, 99.7%). Nafion perfluorinated resin solution (5 wt% in lower aliphatic alcohols and water, contains 15–20% water). Sodium carbonate (Na_2_CO_3_, ≥99%). Isopropanol for HPLC ((CH_3_)_2_CHOH, 99%). All of the materials were purchased from Sigma-Aldrich (Milan, Italy), and they were used as received unless otherwise specified. 

### 2.2. Synthesis of CuZnAl-Oxide Based Catalysts

In this work, catalysts with different Cu, Zn, and Al amounts were synthesized by the co-precipitation method. The first synthesized catalyst (i.e., CuO/ZnO/Al_2_O_3_) contains the following molar concentrations of metal nitrates used as a precursor: Cu:Zn:Al = 0.6 M:0.3 M:0.1 M. Then, to evaluate the contribution of aluminium to the electrochemical performance, a second catalyst, CuO/ZnO, whose concentration of precursors is Cu:Zn = 0.6 M:0.3 M, was synthesized. Finally, we eliminated the Zn for comparative purposes to obtain a third catalyst that was synthesized with a copper nitrate concentration of 0.6 M. For these catalysts, each metal concentration remained constant. Table 1 lists the abovementioned catalysts and their preparation conditions.

**Figure 1:**
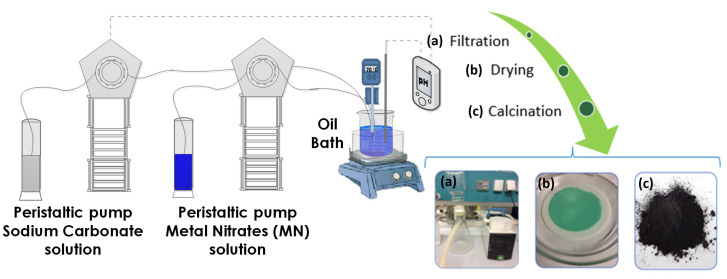
Synthesis process setup.

The catalysts were prepared according to a procedure similar to the one described in the literature [28], by optimizing the synthesis conditions as shown below. The procedure began with the preparation of a solution of hydrated metal nitrates as precursors (Cu(NO_3_)_2_, Zn(NO_3_)_2_, and Al(NO_3_)_3_). This solution was prepared with the concentrations specified in Table 1 for each catalyst. The metal amounts used in the case of the tricomponent catalyst (CuZA-06-03-01) were 1.5 g of Cu (24 mmol), 0.78 g of Zn (12 mmol), and 0.11 g of Al (4 mmol). The metal quantities remained constant for the CuZ-06-03 and Cu-06 catalysts. As a precipitating agent, a solution of Na_2_CO_3_ (1 M) was used. As shown in Figure 1, the setup consists of a beaker with an initial volume of distilled water of 200 mL, immersed in a hot silicone oil bath to maintain the temperature at 70 °C. It is important to mention that, during the precipitation process, the pH and temperature were controlled. The pH was controlled using the MC720 pH Controller. This regulator allows for the setting of the desired pH value (pH = 7 in this case) through a peristaltic pump, which automatically sends the necessary amount of precipitating agent to the beaker to maintain the pH at the desired value. The temperature was monitored with a thermocouple. The synthesis process begins with the pumping of the nitrate solution, whose volume is 40 mL, at a constant flow rate of 5 mL min^−1^, using a peristaltic pump.

The co-precipitation stopped when the 40 mL of metal nitrate solution has been added. The system was placed in an ageing condition for 1 h, then the precipitate was filtered, and left to dry at 60 °C in an oven overnight. Finally, calcination was performed at 350 °C in a muffle for 3 h with a heating ramp of 2 °C min^−1^, as shown in Figure 1. Each synthesis was performed at least 3 times to assess the reproducibility of the process and of the final prepared material.

### 2.3. Characterization of Powder Catalysts

The field emission scanning electron microscopy ZEISS MERLIN FE-SEM (Oberkochen, Germany), equipped with an energy dispersive X-ray spectroscopy system (EDS) operated at 3 kV, was used to obtain the morphological characterization and the contents of the relative element of the samples. The samples were prepared by dispersing a small amount of the particles in Isopropanol through ultrasonic mixing for 30 min, and subsequently placing a drop of the dispersion on a copper grid coated with an amorphous layer of carbon. Finally, the sample was dried at room temperature before the FESEM analysis.

The molar composition of the samples was measured via the inductively coupled plasma (ICP), with an iCAP 7600 DUO (Thermo Fisher Scientific, Waltham, MA, USA) instrument. The chemical attack was carried out in a microwave oven through the following steps: 100 mg of the sample was weighed and solubilized in 10 ml of an acid mixture (6 mL HCl, 2 mL HNO_3_, and 2 mL HF). Then, a thermal treatment at 200 °C for 15 min with a ramp of 10 °C min^−1^ was performed, followed by cooling for about 30 min to ambient conditions. Next, the sample was diluted to ~1 ppm with Milli-Q distilled water and filtered using a 0.45 μm PTFE filter before the ICP analyses.

The transmission electron microscopy (TEM) characterization was performed on an FEI Tecnai G2 F20 S-Twin (Hillsboro, OR, USA) instrument, equipped with an EDAX Si(Li) detector (30 mm^2^ active areas) for EDS spectroscopy. For the preparation of the sample, the powders were dispersed in high-purity ethanol, sonicated for 30 min, and subsequently drop-casted on Au holey carbon TEM grids.

The materials were characterized utilizing N_2_ adsorption to obtain the main textural parameters such as the specific surface area, total pore volume, and the pore size distribution. The N_2_ adsorption/desorption isotherms at 77 K were measured in a volumetric equipment TriStar II 3020 (Norcross, GA, USA) supplied by Micromeritics. All of the samples were previously outgassed at 200 °C for 2 h. The N_2_ adsorption/desorption isotherms were used to evaluate the surface area using the Brunauer–Emmett–Teller (BET) equation. The method of Barrett, Joyner, and Halenda (BJH) was used for the calculation of the pore size distribution from experimental isotherms using the Kelvin model of pore filling.

Information on the crystallinity of the samples was obtained from the X-ray diffraction (XRD) technique. The technique uses a Panalytical X’Pert PRO (Malvern, UK) diffractometer that works in Bragg-Brentano configuration and is equipped with Cu Kα radiation (λ = 1.5418 Å) set at 40 kV and 40 mA. The crystallite sizes were calculated using the Scherrer formula *D = kλ*/*β cos θ*, where *D* is the average crystallite size (nm), λ is the wavelength of X-ray radiation (0.15418 nm), *k* is the shape factor (0.90), and β is the full-width half-maximum, which was corrected for instrumental broadening. The phase purity of the samples was examined by X-ray diffraction in the 2θ range of 20–70° with a scanning step of 0.013°.

The X-ray photoelectron spectroscopy (XPS) measurements were carried out using a PHI 5000 Versa Probe (Physical Electronics, Chanhassen, MI, USA) system. The instrument is equipped with a monochromatic X-ray source of 1486.6 eV (Al K-alpha) to determine the surface composition of the prepared ternary catalysts. All of the core-level peak energies were referenced to the C1s peak at 284.5 eV, and the background signal, in high resolution (HR) spectra, was subtracted with a Shirley function. The deconvolution procedure has been completed using the Multipak 9.7 dedicated software.

### 2.4. Electrochemical Cell and Experimental Conditions

#### 2.4.1. RDE System

**Figure 2:**
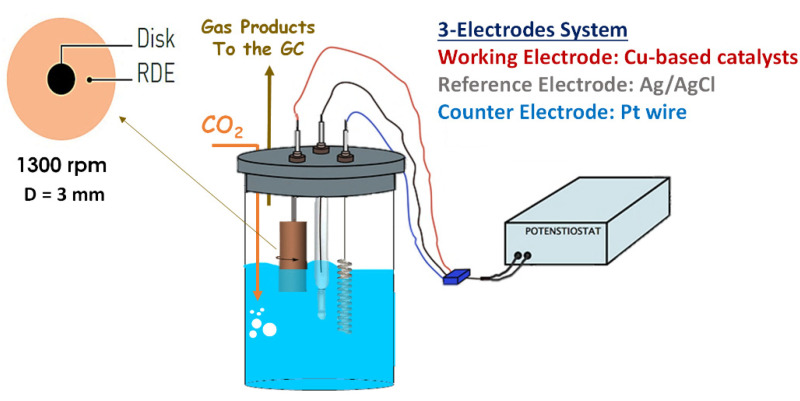
Simplified conceptual scheme of the CO_2_ electrochemical reduction setup.

The electrochemical characterization of the samples consists of testing the catalytic activity (at room temperature and atmospheric pressure) using a traditional three-electrodes electrochemical cell. The CuZnAl-oxide based catalysts were deposited on a glassy carbon rotating disk electrode (RDE), which was used as a working electrode (electrode area of 0.071 cm^2^) to eliminate the effects of mass transfer limitations. The counter electrode was a platinum wire, and the reference electrode was a silver/silver chloride electrode (Ag/AgCl, 3 M NaCl). Similar to the electrolyte, 70 mL of KHCO_3_ (0.1 M) were used (see Figure 2). A Biologic VSP-300 multichannel potentiostat and RRDE-3A rotator system were used to perform the electrochemical tests.

The catalytic inks were prepared by ultrasonically dispersing the catalytic material particles with a Nafion solution used as a binder, and Isopropanol (99% purity Sigma-Aldrich) used as a carrier. The mass ratio of active-phase/Nafion was 70:30 and the Isopropanol/solids mass ratio was 97:3. A percentage of Vulcan XC 72R Carbon (VC, 9.5 wt% of active-phase) was added to improve the conductivity of the catalyst dispersion. Of note, the active phase of the catalyst is considered the copper oxide that was theoretically deposited. The mixture was sonicated for 10 min before the drop-casting process.

A known quantity of each well-dispersed catalyst ink was pipetted onto the glassy carbon electrode to prepare the working electrode. All of the electrodes were maintained with a loading dose of 0.6 mg_CuO_ cm^−2^. Before deposition, the glassy carbon was polished with a 0.05 μm alumina particle suspension on a moistened polishing cloth. The electrode was dried in situ at 400 rpm for 10 min.

The electrochemical behavior of the catalysts was evaluated through cyclic voltammetry (CV) by applying a potential from 0 to −2 V vs. Ag/AgCl (at a scan rate of 30 mV s^−1^) and linear sweep voltammetry (LSV) by applying a potential from 0 to −3 V vs. Ag/AgCl (at a scan rate of 5 mV s^−1^) to evaluate the onset potential of the materials under N_2_ and CO_2_ bubbling into the electrolyte. The electrochemical reduction of CO_2_ was carried out for 2 h with chronoamperometry (CA) under CO_2_-saturated electrolyte to evaluate the selectivity of the catalyst materials. The tests were performed for each catalyst at three different potentials: −1.5, −1.75, and −2 V vs. Ag/AgCl (−0.89, −1.14, and −1.39 V vs. RHE) after carrying out a pre-reduction in N_2_-saturated 0.1 M KHCO_3_ aqueous solutions at −1.5 V vs. Ag/AgCl (−0.89 V vs. RHE). The CO_2_ flow rate was set via a mass flow controller (EL-Flow Select, PN64) at 16.29 NmL min^−1^. All of the electrochemical measurements were made at a rotation speed of 1300 rpm.

#### 2.4.2. H-Cell System

**Figure 3:**
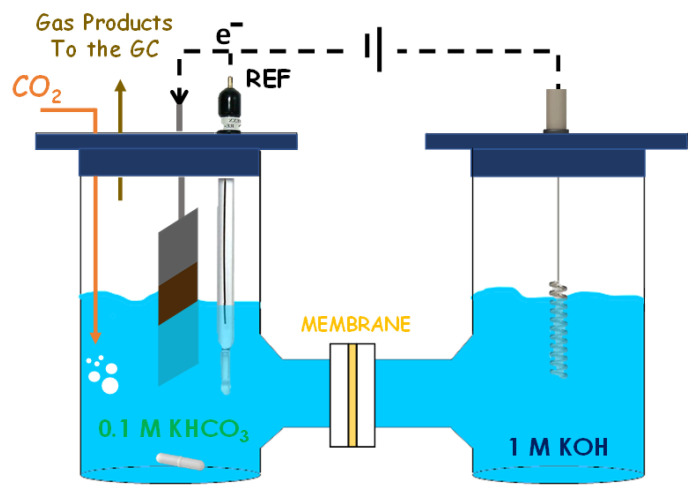
Simplified conceptual scheme of the electrochemical CO_2_ reduction in H-cell setup.

The electrodes of the CuZA-06-03-01 catalyst (geometric area, A = 1 cm^2^) were prepared by airbrushing a suspension of the material onto a porous Toray carbon paper (TP-060T Quintech, Göppingen, Germany). The catalytic ink was produced with the recipe mentioned above. The catalyst loading was 0.6 mg_CuO_ cm^−2^, as in the case of the RDE system. The counter electrode was a platinum wire, and the reference electrode was a silver/silver chloride electrode (Ag/AgCl, 3 M NaCl). As in the previous case, the CO_2_ flow gas was purged into the cathode chamber, and gas-phase products are collected from the headspace. The electrodes were pre-reduced in a N_2_-saturated 0.1 M KHCO_3_ aqueous solution for 30 min at −1.5 V vs. Ag/AgCl (−0.89 V vs. RHE) before each electrochemical measurement. The co-electrolysis of CO_2_ was carried out in the cathodic compartment for 2 h in a CO_2_-saturated 0.1 M KHCO_3_ aqueous solution at two different current densities: −12 and −24 mA cm^−2^. The catholyte was stirred to try to imitate the RDE system conditions and to avoid mass transfer limitation problems. In the anodic compartment, 1 M KOH was used as the electrolyte to decrease the electrolyte resistance issues. The cathodic and anodic compartments were separated by a Nafion 117 membrane (Figure 3). These tests were carried out to investigate the effect of the morphology and crystalline phase changes on the electroactivity of the tricomponent electrodes. 

In both cases, the concentration of the outgoing gases was analyzed continuously through a gas chromatograph (Inficon—Micro GC Fusion Gas Analyzer, Budrio, Italy) equipped with two channels with a 10 m Rt-Molsieve 5 A column and an 8 m Rt-Q-Bond column, respectively, and micro thermal conductivity detectors (micro-TCDs). In contrast, the concentration of the liquid products was analyzed with (1) a high-performance liquid chromatograph (HPLC) Prominence by Shimadzu, (Milan, Italy), equipped with two detectors: RID-10A and PDA 212 nm, a Rezex ROA Organic acid column (D: 7.8 mm; L: 300 mm) and using 5 mM H_2_SO_4_ as the mobile phase; (2) a gas chromatograph with a mass spectrometer detector (Perkin Elmer GC, Clarus 580, Milano, Italy) equipped with a Head Space and a Stabilwax-DA column. Electrochemical impedance spectroscopy (EIS) measurements were performed at various potentials from −0.75 to −2 V vs. Ag/AgCl with an AC signal of 10 mV of amplitude and 10^−1^–10^4^ Hz of the frequency range in a CO_2_-saturated 0.1 M KHCO_3_ aqueous solution. The changes in electrolyte pH values (i.e., 8.4 in the solution saturated with N_2_ and 6.8 in the presence of CO_2_) are considered. For this reason, the applied potentials will be represented versus the RHE in Section 3 using Equation (11).

(11) $$E_{RHE}=E_{Ag/AgCl}+0.21+0.059\;\cdot \;pH\;$$

The relative formation of a determinate product was calculated through the Faradaic efficiency (FE), which describes the selectivity of a catalyst. FE is defined as the ratio of the number of coulombs required to form a certain amount of product (determined by the chemical analysis) to the total charge over a specific time interval [6]. The Faradaic efficiency was calculated according to Equation (12).

(12) $${\mathrm{FE}}_{i}=\frac{z\cdot F\cdot \left({\dot{n}}_{i,\mathrm{out}}\right)}{j\;\cdot A\;\cdot t}$$

where *z* is the number of electrons required to produce a determined product, F is the Faraday constant (96,485 C mol^−1^), j is the current density (A m^−2^), A is the electrode area (m^2^), t is the reaction time (s), and ${\overset{\text{˙}}{n}}_{i,\mathrm{out}}$ is the outlet molar flow rate of each product.

## 3. Results and Discussion

### 3.1. Physico-Chemical Characterization of Synthesized Powder Catalysts

**Figure 4:**
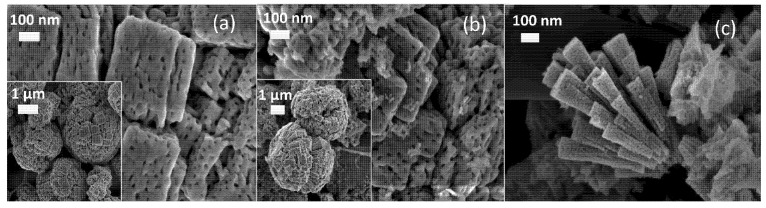
FESEM micrographs of CuZnAl-oxide based catalysts (**a**) Cu-06, (**b**) CuZ-06-03, (**c**) CuZA-06-03-01.

The FESEM micrographs of the synthesized catalysts are shown in Figure 4. The metal precursor solution influences the final morphology of these samples. However, their structures grow from a common nucleation point. In particular, the Cu-06 catalyst presents spherical hierarchical microstructures formed by porous pyramids (Figure 4a). The relative composition of the particles was analyzed by EDS measurements in three different zones of the sample, resulting in a Cu/O atomic ratio of 1:1, as shown in Table 2. When Zn was added into the precursor solution to produce the CuZ-06-03 catalyst, no changes were observed in the hierarchical structure, but very small ZnO (ca. < 20 nm) clusters were detected on the spherical surface, as shown in Figure 4b. In this case, EDS revealed that these structures are copper-rich (atomic ratio Cu/Zn = 60/40, within a range of ±0.5 mol%). In contrast, also adding Al into the precursor solution promotes a ternary CuZA-06-03-01 catalyst that is mainly constituted by a porous corsage of nano-pyramidal structures. Figure 4c shows the formation of small particles at the surface of these pyramid-shaped particles, along with agglomerates of snowflake-shaped small crystals. The Cu/Zn/Al metal ratio in the resulting catalyst corresponded to the metal ratio settled by the precursor solution concentrations, i.e., 60/30/10 mol% (within a range of ±3 mol%), as in the original synthesis procedure [28]. However, the controlled nano-pyramidal morphology is here presented for the first time. It is believed that the appropriate control of the pH and mixing with automatized systems play an important role in controlling the homogeneity of the synthesis media and the reproducibility of the obtained nanostructures.

The total pore volume increased after incorporating metal oxides (ZnO and Al_2_O_3_), as demonstrated by the nitrogen uptake increase (as shown in Table 2), which indicates a transition from narrow porosity to a wide pore size distribution.

The Cu-06 showed a narrow distribution of pore diameters with a mean value of 25 nm. In contrast, the CuZ-06-03 and CuZA-06-03-01 catalysts showed a trimodal and bimodal pore size distribution, showing peaks centred at 10–23–31 nm and 7–15 nm, respectively (see Figure S1 in SI). In agreement with this observation, the ternary catalyst presented the highest BET surface area: The Zn and Al-based metal oxides provoked a better development of the mesoporous structure [29]. The presence of Cu, Zn, and Al was further confirmed via the ICP analysis. Nevertheless, the molar composition detected by the analysis is slightly different from the EDS (Table 2), as shown in Table 3.

A relevant feature from the N_2_ isotherms (see Figure S2 in SI) is that all of the curves are of type III, convex to the X-axis and do not present a monolayer formation. It indicates that the adsorbent-adsorbate interactions are relatively weak, and the adsorbed molecules are clustered around the most favorable sites on the surface of a non-porous solid. Moreover, the isotherms presented a hysteresis loop in the desorption branch. The presence of slit-shaped mesopores gives origin to loops of this type [29]. It is in agreement with the pore size distribution mentioned above.

**Figure 5:**
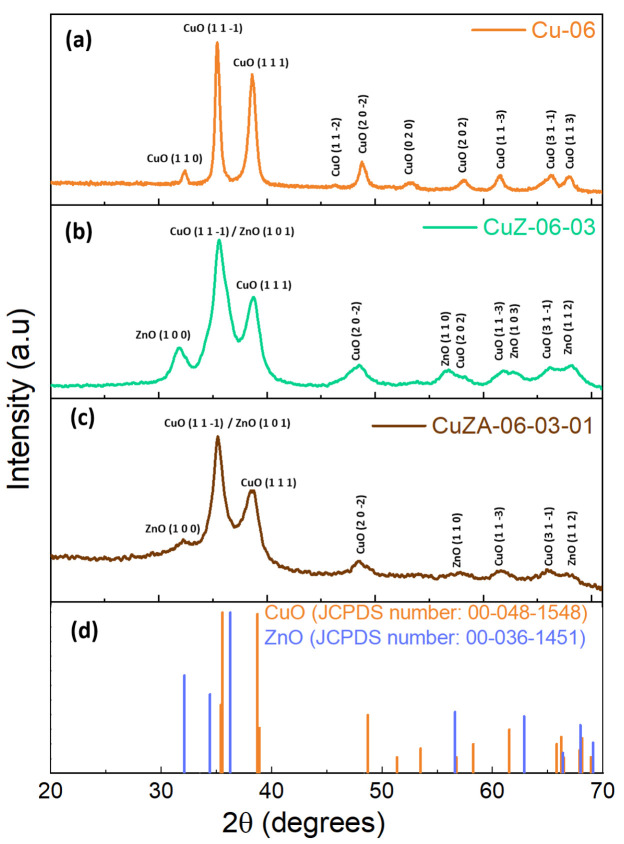
XRD patterns of CuZnAl-oxide based catalysts (**a**) Cu-06, (**b**) CuZ-06-03, (**c**) CuZA-06-03-01, and (**d**) the JCPDS card plot of CuO and ZnO.

The XRD patterns of the powders are shown in Figure 5. On the Cu-06 catalyst (Figure 5a), the defined reflections can be assigned to only the monoclinic CuO crystalline phase, in particular to the (110), (11-1), (111), (11-2), (20-2), (020), (202), (11-3), (31-1), and (113) planes (JCPDS number: 00-048-1548). Similar crystalline phases are observed on the CuZ-06-03 and CuZA-06-03-01 samples, which are composed of monoclinic CuO and hexagonal ZnO (JCPDS number: 00-036-1451). The Al_2_O_3_ diffraction peaks are not visible since they probably remain as an amorphous phase. This could be due to the low calcination temperature used in this work during the synthesis of the prepared samples since the formation of Al_2_O_3_ crystalline species is unlikely at 350 °C [30]. However, the presence of Al is confirmed by the ICP analysis (Table 3). It is interesting to note that not all of the CuO reflections shown in the Cu-06 catalyst are observed on CuZ-06-03 and CuZA-06-03-01. Simultaneously, the crystalline planes corresponding to ZnO are not the same between CuZ and CuZA. The more significant difference is associated with the broadening of the diffraction peaks. It was observed that the broader diffraction peaks were produced from the catalysts after incorporating Zn and/or Al in the precursor, indicating that the addition of these metals inhibits the growth/crystallization of the final CuO phases, forming smaller crystallites of the latter metal oxide. This fact agrees with the crystallite size of CuO, corresponding to the (11-1) facet, calculated from the Debye-Scherrer equation (see Table 2).

**Figure 6:**
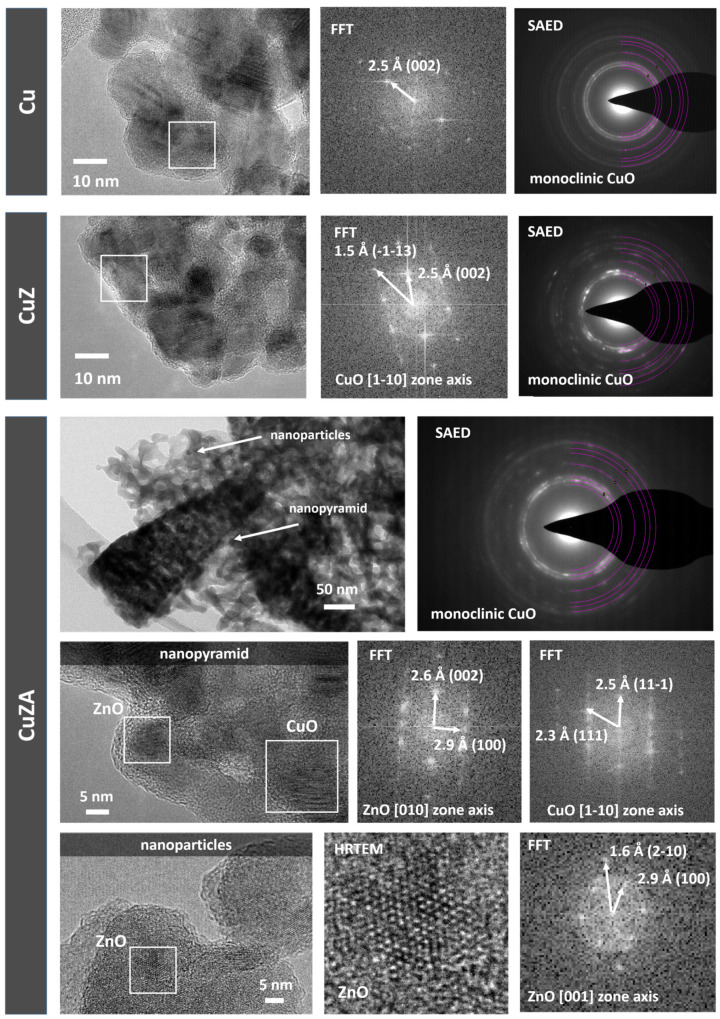
TEM analysis for samples Cu-06, CuZ-06-03, and CuZA-06-03-1. Fast Fourier transforms (FFT) of the highlighted regions are provided, alongside selected area electron diffraction (SAED) patterns and a high-resolution TEM (HRTEM) image of a ZnO crystalline domain in sample CuZA-06-03-01.

The crystalline structure at the nanoscale was further characterized by transmission electron microscopy, whose main results are presented in Figure 6.

For the Cu-06 and CuZ-06-03 samples, it was possible to determine the electron-transparent regions that consist of porous aggregates of nanoparticles, allowing for both the selected area electron diffraction (SAED) analysis and high-resolution TEM imaging (HR-TEM). For the results of electron diffraction, for both kinds of samples, a polycrystalline monoclinic CuO phase is identified (see Figure S3 and Table S1 in the Supporting Information for a detailed description of the analysis).

The absence of a clear indication of the ZnO phase in the CuZ-06-03 sample could be explained by a possible overlapping of these diffraction rings with the ones associated to the CuO phase, alongside an intrinsically lower number of analyzed crystallites with respect to XRD. The analysis of HR-TEM images and the corresponding Fast Fourier transforms (FFT) confirm the presence of CuO crystallites with a nanometric size for both the Cu-06 and CuZ-06-03, in accordance with the previously discussed XRD results obtained from the Scherrer equation. It must be stressed that, although it was not possible to clearly identify the ZnO crystallites in the CuZ-06-03 sample, the successful incorporation of Zn in the studied regions is confirmed by the EDS analysis (see Figure S4 in SI).

The CuZA-06-03-01 sample clearly shows two different morphologies according to the FESEM results: Aggregated nanoparticles and porous nanopyramids. Herein, electron diffraction confirms the presence of the monoclinic CuO phase (see the Supporting Information for details), but it does not provide information on the ZnO phase. However, HR-TEM images give further insight into the crystalline structure of the sample for both nanoparticle aggregates and nanopyramids. Specifically, it is possible to identify the simultaneous presence of monoclinic CuO and hexagonal ZnO crystallites at the edge of the nanopyramids. It is also worth noticing a clear amorphous layer on the external surface that could be ascribed to the alumina phase, which is not supposed to crystallize at the calcination temperatures used in this work.

For the aggregated nanoparticles, extremely small crystalline domains (<5 nm in size) are identified, which can be attributed to the hexagonal ZnO phase. However, the ZnO nanoparticles are predominantly amorphous, as confirmed by HR-TEM imaging. The successful incorporation of Zn and Al in both nanoparticles and nanopyramids is proven by the EDS elemental maps, such as the one provided in the Supporting Information (Figure S4). Moreover, a careful comparison of the elemental contrast in the EDS maps reveals that the concentration of Al and Zn is higher in the predominantly amorphous nanoparticles with respect to the nanopyramids.

**Figure 7:**
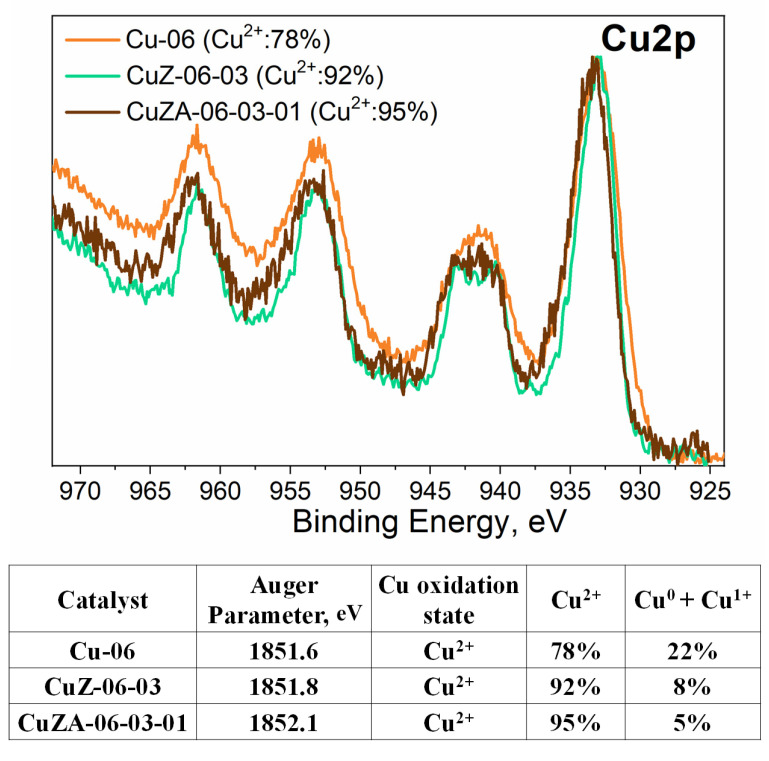
The Cu2p doublet region acquired in high-resolution mode, and the table shows the percentage of oxidation states of copper calculated from Auger parameter values on the surface of the CuZnAl-oxide based catalysts.

The XPS measurements were performed on the synthesized samples to investigate the chemical composition of their surface. The Cu2p doublet spectra, determined in the high-resolution mode, display a typical spectrum mainly related to CuO, which is clearly visible thanks to the presence of the Cu^2+^ satellite at 940–945 eV (see Figure 7) [31]. The Cu^2+^ state has been already observed in bulk with the XRD analysis. Moreover, the Auger CuL_3_M_4.5_M_4.5_ region was acquired to obtain additional details since the deconvolution procedure of the Cu2p spectra results is complex due to the overlapping of binding energies of the different Cu oxidation states. The table in Figure 7 shows the modified Auger parameters of the samples with an average value (1851.8 ± 0.3 eV) that is typical of Cu^2+^ species, indicating that their surfaces are mainly composed of CuO, within a thickness of at least 5–10 nm (the sensible depth for XPS).

Furthermore, the precise amount of each oxidation state of copper was estimated through the method developed by M. Biesinger et al. [31]. Therefore, it was possible to evaluate the ratio between Cu^2+^ and Cu^0^ + Cu^+^ by fitting the Cu2p_3/2_ peak and its related satellite. After applying this procedure, the values reported in Figure 7 were obtained, from which it is possible to notice that the Cu-06 catalyst presents a higher percentage of Cu^0^ + Cu^1+^ (22%) than the other catalysts (<10%).

**Figure 8:**
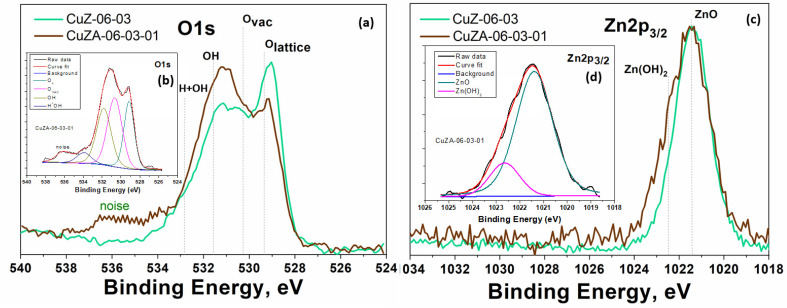
XPS spectra of CuZ-06-03 and CuZA-06-03-01 catalysts: (**a**) High-resolution O1s XPS spectra, (**b**) inset with deconvolution peaks of the O1s spectra, (**c**) high resolution Zn2p3/2 XPS spectra, and (**d**) inset with deconvolution peaks of the Zn2p3/2.

To better understand the characteristics of the mixed-metal oxides samples, the high-resolution XPS spectra for the O1s and Zn2p_3/2_ binding energy regions obtained for the CuZ-06-03 and CuZA-06-03-01 synthesized catalysts are shown in Figure 8. The deconvolution procedure that was applied to the O1s spectrum (not reported) reveals that the O species are divided into the following (Figure 8a): Oxygen into the lattice structure, oxygen vacancies, and oxygen due to adsorbed molecules. The O1s peak at 529.2 eV is assigned to oxygen in the lattice environment, the peak at 530.3 eV is attributed to the oxygen vacancies, while the peaks at 531.6 and 532.9 eV are assigned to weak basic sites on the surface: OH and H*OH, respectively, which are mainly present in the CuZA-06-03-01 catalyst. The presence of oxygen vacancies brings about free electrons, which are shallow electron donors that could enhance the conductivity of a semiconductor material such as CuO. Meanwhile, the binding energy of the Zn2p_3/2_ peak at around 1021.2 eV indicates that Zn is present as ZnO in both catalysts (see Figure 8b), while the peak at 1022.5 eV confirms the presence of Zn(OH)_2_ species, which corroborates the alkalinity of the surface of the CuZA-06-03-01 catalyst again. As expected, the addition of ZnO and Al_2_O_3_, as amphoteric supports, enhanced the surface basicity [32,33,34], improving the catalytic activity and the adsorption of CO_2_/CO molecules.

### 3.2. Physico-Chemical Characterization of the Prepared Electrodes

**Figure 9:**
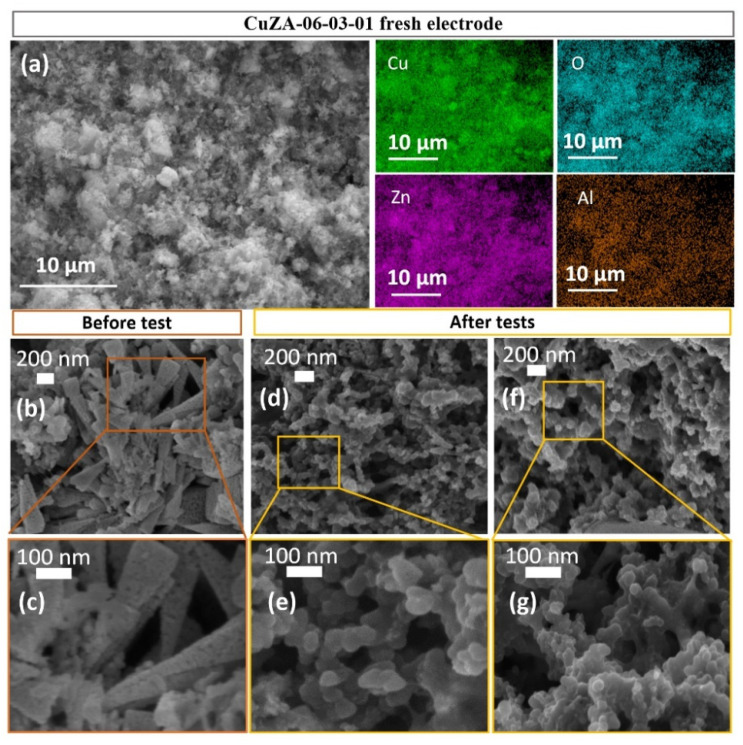
FESEM-EDS analyses of CuZA-06-03-01 GDEs: (**a**) Low magnification FESEM image and EDS maps of Cu, O, Zn, and Al elements on the fresh electrode; FESEM images before (**b**,**c**) and after (**d**–**g**) electrochemical CO_2_ reduction tests in the 0.1 M KHCO_3_ electrolyte at −12 mA cm^−2^ (**d**,**e**) and −24 mA cm^−2^ (**f**,**g**).

As a representative of the tested electrodes, Figure 9a shows a low magnification FESEM image with an EDS map of Cu, O, Zn, and Al elements in the CuZA-06-03-01 deposited layer, which demonstrates an uniform coverage of these elements on the carbon paper substrate. Other elements (i.e., C, F, and S, not shown) were also visible in the EDS analyses due to the carbon substrate and the binder (Nafion) used for the catalytic ink preparation.

Figure 9b,c shows a high magnification FESEM image, which evidences the porous corsage of nano-pyramidal structures of the tricomponent material. The Cu/Zn/Al ratio found at 62:31:7 was practically the same as in the powder catalyst. On the contrary, Figure 9d–g shows the modified nanostructured porous morphology of the CuZA-06-03-01 particles, confirming the phase composition modification after testing. In fact, the crystalline structures, shown in Figure S5 in SI, evidence that the bulk of the CuZA-06-03-10 tested electrodes has changed during the co-electrolysis of CO_2_.

### 3.3. Electrochemical Measurements

#### 3.3.1. Cyclic Voltammetry Characterization of CuZnAl-Oxide Based Electrodes

**Figure 10:**
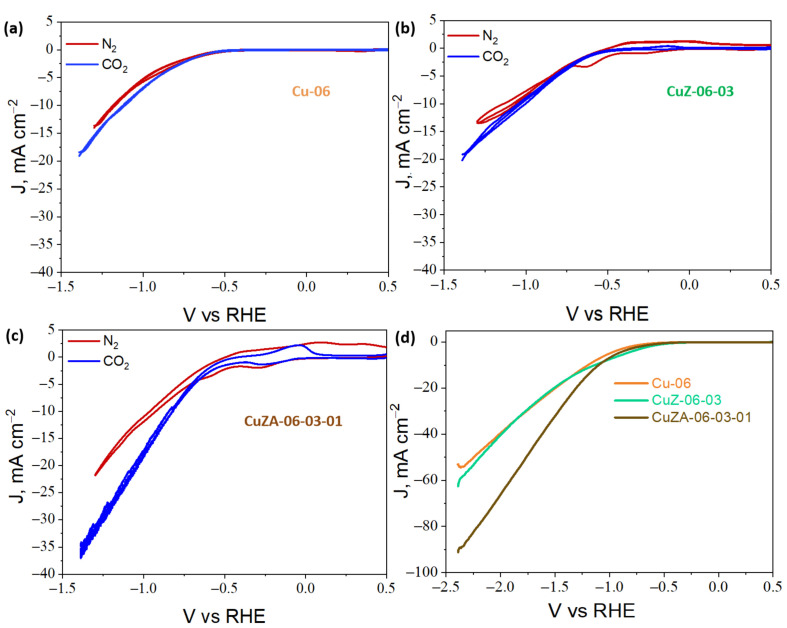
Cyclic voltammetry responses for (**a**) Cu-06, (**b**) CuZ-06-03, (**c**) and CuZA-06-03-01 electrodes in a CO_2_ and N_2_ saturated 0.1 M KHCO_3_ aqueous solution. Linear polarization curves obtained for the CuZnAl-oxide based electrodes in the CO_2_-purged 0.1 M KHCO_3_ electrolyte (scan rate: 5 mV s^−1^) (**d**).

Before the ER-CO_2_, the system was initially degassed for 20 min with N_2_ at a flow rate of 50 mL min^−1^. Then, the electrodes were pre-reduced for 30 min at −1.5 V vs. Ag/AgCl (−0.89 V vs. RHE) before each electrochemical measurement. Next, a blank CV was performed on the N_2_-purged electrolyte by scanning the electrode from 0.5 to −1.4 V vs. RHE to study the performance of the CuZnAl-oxide based catalysts in the working solution. The same process was repeated in the CO_2_-saturated electrolyte after bubbling CO_2_ for 30 min with a flow rate of 50 mL min^−1^. Figure 10a–c shows the reduction/oxidation features of the catalysts: The red and blue curves represent the CVs carried out in N_2_ and CO_2_, respectively.

In the case of the Cu-06 and CuZ-06-03, the CVs demonstrate that there is a slight increase of the activity in the presence of CO_2_ with respect to N_2_, which may be associated with CO_2_ adsorption on the electrodes and further reaction with H^+^ (see Figure 10a,b). Figure 10c shows that this trend is more pronounced in the case of the CuZA-06-03-01 sample, for which the electroreduction response can be mainly attributed to the CO_2_ conversion (blue curve) at potentials more negative than −0.6 V vs. RHE. In Figure 10, the other three main characteristics can also be seen. First, the CuZ and CuZA catalysts have a capacitive behavior in the presence of N_2_ (see Figure 10b,c), although Vulcan Carbon was added to increase the electrode conductivity. The formation of this electric double layer between the surface of the catalyst and the electrolyte solution near the electrode is not present under the CO_2_-purged electrolyte. This capacitive effect can be attributed to the partial reduction of the catalyst (Cu^2+^ to Cu^1+^ and/or Cu^1+^ to Cu^0^) during the experiments under N_2_ flow. It makes the material more conductive [35,36]. The second characteristic is that the CuZ and CuZA catalysts present redox peaks, which can be associated with oxidation (positive currents) or reduction (negative current) processes. The anodic-cathodic peaks in the current-potential curve under N_2_ are possibly associated with the partial formation/reduction of the oxides on the electrode surface (red curves).

On the other hand, these branches in the CV under CO_2_ could be associated with reduction reaction intermediates adsorbed on the catalyst surface and consequently, to the oxidation of these adsorbed species [35,37] (blue curves). It was determined by the method proposed by Elgrishi, N. et al. [38] that the adsorption of CO_2_ reduction species is the prevalent phenomenon causing those peaks (see the results reported in Figure S6 in the SI). These latter anodic-cathodic branches have higher current densities in the CuZA-06-03-01 CO_2_-curve than in the CuZ-06-03 one. These results agree with the XPS measurements shown in Figure 8, in which the increased alkalinity proportioned by the basic sites of ZnO and/or Al_2_O_3_ pairs on the catalyst surface serve to promote the adsorption of CO_2_ and its reaction intermediates [33,34]. Moreover, this relevant characteristic is associated with the higher total current density, in the presence of CO_2_, obtained with the CuZA-06-03-01 electrode than with the other ones (Figure 10c), which seems to have a correlation with the role of the mixed-metal oxides in promoting CO_2_ adsorption and conversion. Furthermore, this is observed in the linear polarization curves shown in Figure 10d, where an industrially-relevant maximum current density (the total activity of the electrode) of 90 mA cm^−2^ was recorded with the CuZA-06-03-01 electrode, which is a worth noting value in view of the practical application of this new electrocatalyst.

In this context, early DFT calculations have demonstrated that two uphill reactions limit the activity of catalysts for the ER-CO_2_: (1) The formation of *CO through CO_2_ activation and hydrogenation is the rate determining step (RDS) and (2) the hydrogenation of as formed *CO is the selectivity determining step (SDS) [39,40,41]. Therefore, the surface basicity of the ternary CuZA catalyst could promote the RDS of CO_2_ activation and formation of the *CO intermediate, improving the catalytic electroactivity of the sample.

#### 3.3.2. Electrochemical Impedance Spectroscopy

**Figure 11:**
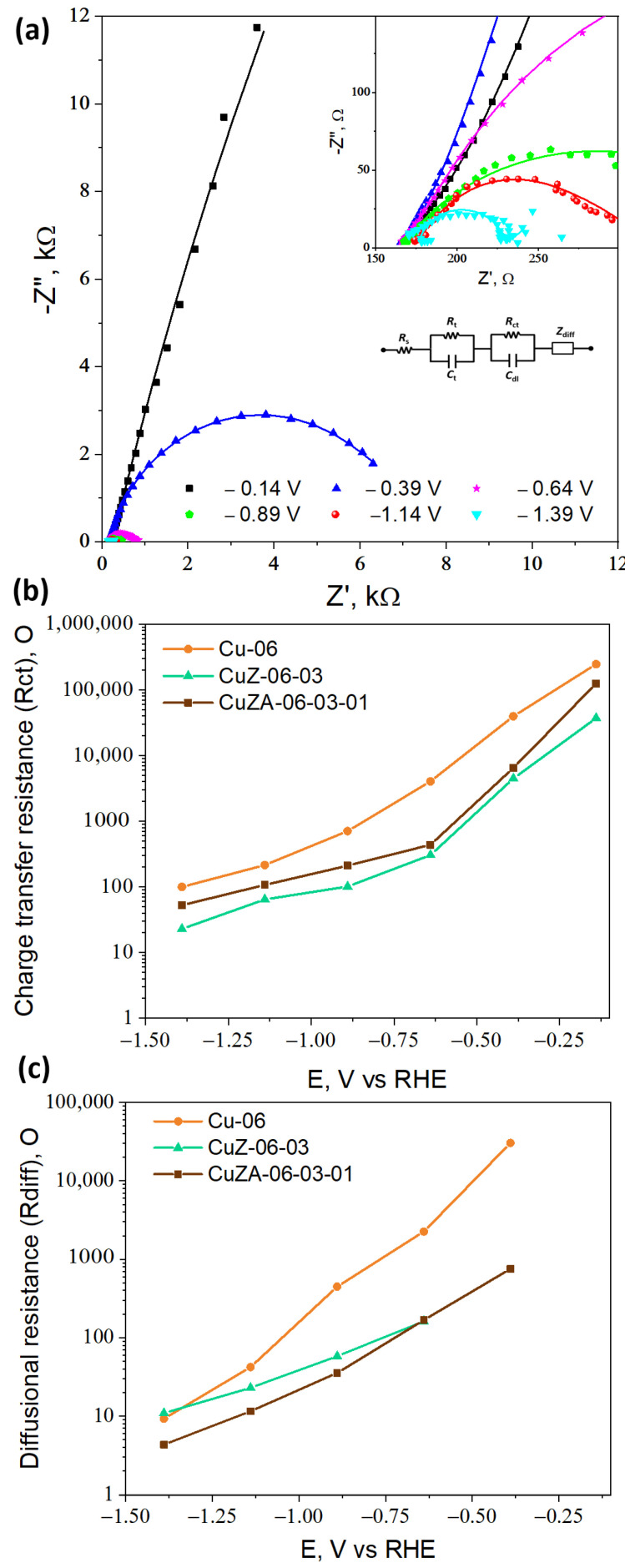
(**a**) Nyquist plots related to the CuZA-06-03-01 electrode measured at different potentials (V vs. RHE) in the CO_2_-purged electrolyte (the points are experimental data; the set of numbers are the curves calculated through a fitting procedure using the equivalent electrical circuit shown in the inset of (**a**)). (**b**) Charge transfer resistances and (**c**) diffusional resistances reported as a function of the potential of the CuZnAl-oxide based electrodes.

To further investigate the effect of the addition of ZnO and Al_2_O_3_ on the charge transfer and transport properties of the resulting catalyst during the electroreduction reactions, EIS was carried out in a CO_2_-purged 0.1 M KHCO_3_ electrolyte. Figure 11a shows the Nyquist plots for the CuZA-06-03-01 electrode measured at different potentials. At more negative potentials, the impedance module value decreases, indicating higher Faradaic reaction rates [42]. The same behavior was observed for the CuZ-06-03 and Cu-06 samples (data not shown).

The experimental curves were fitted through the equivalent electrical circuit shown in the inset of Figure 11a. The curves were composed of a series resistance *R*s (associated with the electrolyte resistance), a parallel between the resistance *R_t_* and the capacitance *C_t_* (representing the charge transport inside the catalyst layer), another parallel *R_ct_*//*C_dl_* (related to the charge transfer at the electrode/electrolyte interface), and the Warburg impedance *Z_diff_* (related to the diffusion properties of reagents and products in the electrolytic solution) [43]. The calculated curves (solid lines) at different potentials for the CuZA-06-03-01 electrode are superimposed to the measured data in Figure 11a: The fitting also resulted well for all of the other tested electrodes. The *R_s_*, *R_t_*, *C_t_*, and *C_dl_* parameters have negligible dependence on the potential, and the obtained values are reported in Table 4.

The electrochemically active surface area (ECSA) was calculated according to Equation (13), where $C_{ref}$ is the double-layer capacitance of a flat reference surface.

(13) $$ECSA\;=\;\frac{C_{dl}}{C_{ref}}$$

A value of 28 µF was used as the reference value for a flat Cu electrode surface in this work [42]. The highest ECSA of the mixed-metal oxide electrodes could be attributed to their smaller crystallites with respect to the Cu-06 sample (see Table 2). It is worth mentioning that the addition of Al_2_O_3_ was not very successful in increasing the ECSA as in the case of the only addition of ZnO to the Cu-based catalyst. In fact, Al_2_O_3_ was effective in increasing the BET surface area (Table 2), but not the electrochemical active one. However, the CuZA-06-03-01 electrode is characterized by the lower charge transport resistance *R_t_*, implying that the uniform distribution of all the elements constituting this material and its open nanosized structure is successful in guaranteeing a good electron conductivity of the catalytic layer, which contains not only the catalyst but also a well dispersed and highly conductive Vulcan carbon.

On the contrary, the charge transfer resistance *R_ct_* and diffusional mass transfer resistance *R_diff_* (calculated from *Z_diff_* employing the formula *Z_diff_* = *R_diff_* (*ω*_d_/j*ω*)^1/2^[tanh(j*ω*/*ω*_d_)^1/2^], with the *ω*_d_ characteristic diffusional frequency [43] are quite sensitive on the potential. The obtained values for all of the CuZnAl-oxide based electrodes are reported in Figure 11b,c. The lowest *R_ct_* characterizes the CuZ-06-03 catalyst at all of the studied potentials (see Figure 11b). This trend agrees with the much larger double-layer capacitance (*C_dl_*) and consequently, the much higher ECSA of CuZ-06-03 than the other two electrodes. On the other hand, the CuZA-06-03-01 presented the lower *R_diff_* at all of the potentials, as reported in Figure 11c. Therefore, the diffusion of the reagents and products is less hindered on this material. In this case, the intermediates have no struggle to come out of the porous structure of the material and therefore, they can favor products with fewer carbon atoms (e.g., CO). The *R_diff_* behavior agrees with the total pore volume of the as-synthesized catalysts reported in Table 2 (CuZA-06-03-01 > CuZ-06-03 > Cu-06). Consequently, the CuZA-06-03-01 catalyst is characterized by the best transport and diffusional properties, which contribute to the highest activity of this material among the three electrodes, despite its lower ECSA with respect to the CuZ-06-03.

#### 3.3.3. Electrochemical CO_2_ Reduction in the Aqueous Phase

**Figure 12:**
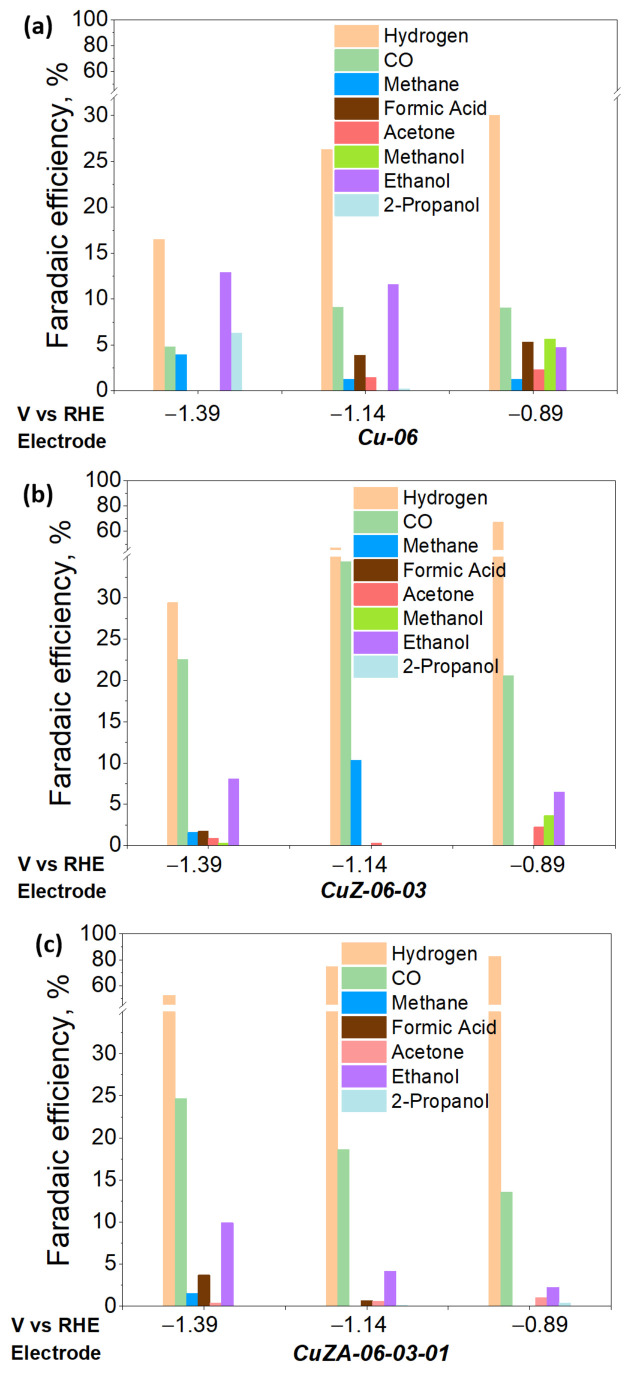
Faradaic efficiencies for CO_2_ reduction products on Cu-06 (**a**), CuZ-06-03 (**b**) and CuZA-06-03-01 (**c**) electrodes at −1.39, −1.14 and −0.89 V vs. RHE.

To study the influence of CuZnAl-oxide based electrodes for the ER-CO_2_, CA measurements under the CO_2_-saturated electrolyte were performed in a RDE system at three different potentials (−1.39, −1.14, and −0.89 V vs. RHE) for 120 min, under the same reaction media. The resulting products were analyzed by the online micro-GC (hydrogen, CO, and methane), HPLC (formic acid), and GC-MSD (acetone, methanol, ethanol, 2-propanol, and other oxygenates such as acetaldehyde, propionaldehyde, etc.) analysis. The Faraday efficiencies for each CO_2_ reduction product were calculated according to Equation (12), and the resulting values are presented in Figure 12.

The selectivity towards the formation of hydrocarbons and multicarbon oxygenates (including aldehydes and alcohols) is higher in the Cu-06 electrode than for the other Cu-based electrodes containing Zn and/or Al oxides [36,44]. Recent studies have shown that the interface between the biphasic copper oxidation states (i.e., Cu^+2^ and/or Cu^+1^ and Cu^0^) contributes to the dimerization of *CO (CO adsorbed on the electrode surface) to generate C-C bonds and C_2+_ products. Therefore, the different copper oxidation states already present in the as-synthesized Cu-06 catalyst and formed during the CV stabilization in N_2_ flow could justify the diverse selectivity of this electrode [19,45]. Moreover, in good agreement with previous studies [18,46,47], hydrocarbons and multicarbon oxygenates are favorably produced on Cu nanoparticles higher than 15 nm, whereas CO and H_2_ are favored on smaller Cu particles (<3–6 nm). Therefore, the difference in the CuO crystallite size of the as-synthesized catalysts (i.e., 16, 7, and 8 nm for the Cu-06, CuZn-06-03, and CuZnAl-06-03-01, respectively, see Table 2) could explain in a first instance the different selectivity of these electrode materials. Additionally, it should be noted that polycrystalline copper generally catalyzes the formation of a large number of CO_2_ reduction products [48], which is in good agreement with the morphology and behavior of the Cu-06 studied catalyst. Moreover, it could explain the minor amounts of C_2+_ products observed in the other two catalyst materials.

On the other hand, as already observed for a thermocatalytic system, adding metal oxides (ZnO and Al_2_O_3_) to Cu-based catalysts increases the catalyst surface area, contributes to the enhancement of the CO_2_ adsorption on the catalyst surface, and is useful in tuning the binding energies of *CO, *CHO (or *COH) intermediates, favoring C_1_ products such as CO and methanol [49,50]. Accordingly, the here synthesized CuZ-06-03 and CuZA-06-03-01 catalysts are both more selective towards H_2_ and CO formation than the Cu-06 catalyst, which agrees with the low binding energy of CO into Cu- and Zn-based nanocrystals smaller than 15 nm [51,52]. Indeed, the here reported catalyst materials have CuO and ZnO crystal sizes between 7 and 11 nm (see Table 2). In addition, the higher HER activity of these catalysts (see Figure 12) might be due to the poor coordination of ZnO sites, which favor the water adsorption and dissociation reaction [47]. Moreover, it agrees with the water sensitivity of CZA catalysts in the thermocatalysis process [53]. Indeed, the HER is more striking on the CuZA-06-03-01 electrodes, which is correlated to the behavior of alumina-supported catalysts currently employed to reform hydrocarbons suitable for H_2_ production by partial dehydrogenation reactions [54].

The CuZ-06-03 electrode displayed a higher selectivity for CO_2_ reduction products (FE ~ 45% at −1.14 V) than the CuZA-06-03-01 one (FE ~ 25% at −1.14 V), as shown in Figure 12b,c. It could be attributed to the diffusion properties of as-synthesized catalysts shown in Figure 11c. The higher the *R_diff_*, the more hindered the motion of reagents, intermediates, and products. On the contrary, in higher pore volume structures, as in the case of CuZA-06-03-01 electrode (see Table 2), the residence time of CO_2_ reaction intermediates such as CO is possibly lower. Therefore, the CO desorption and transfer outside of the catalytic layer is more feasible than its re-adsorption for C-C bonds formation by *CO dimerization reactions [55,56]. It implies that the higher selectivity for syngas (H_2_ and CO mixtures) production on the ternary tested electrode could be related to its lower diffusional resistance and textural properties. In addition, the selectivity towards syngas of the mixed-metal oxides electrodes could be associated with the low percentage of Cu^0^ and Cu^1+^ on the surface of the pristine material (see Figure 7), which suggest higher stability of the Cu^+2^ species under the electrochemical reduction conditions, due to the CuO stabilization by the well-dispersed ZnO and Al_2_O_3_ structures (as demonstrated by the TEM analysis, see Figure S4 in SI).

It is worth mentioning the dependence of product efficiencies on the applied potential, in which H_2_ was predominantly generated at less negative potentials. In addition, a range of different products was generated at more negative ones. In literature, it has been hypothesized that HER occurs faster at less negative potentials [57]. Therefore, an increase in the applied potential (increase of the reaction kinetics) favors the CO_2_ reduction reaction pathways, implying more than two proton-coupled-electron-transfer processes.

Figure 12 shows that, unlike what has been seen in thermocatalysis for which mixed-metal oxides are used as catalysts to promote methanol production, in electrocatalysis (under the here reported working conditions), the CuO mixed with amphoteric metal oxides, i.e., ZnO and Al_2_O_3,_ promotes CO and H_2_ formation. However, a small percentage of methanol (<10%) was detected at the lowest applied potential (−0.89 V vs. RHE) when Cu-06 and CuZ-06-03 were used as electrodes. It has been proven in a thermocatalytic and electrocatalytic process that Cu^0^ crystals of size > 28 nm are more selective for methanol and alcohols production [9,58]. However, that is not the most probable case in the present work, in which the CuO crystal size of the here prepared Cu-06 and CuZ-06-03 catalysts are 16 and 7 nm, respectively [33,34,59]. However, the co-presence of different Cu oxidation states (Cu^+2^, Cu^+1^, and Cu^0^) on these catalyst materials might play an important role in the alcohol’s productions. Indeed, ethanol was detected at almost all of the applied potentials with all of the tested electrodes, and it was predominantly generated at more negative potentials.

**Figure 13:**
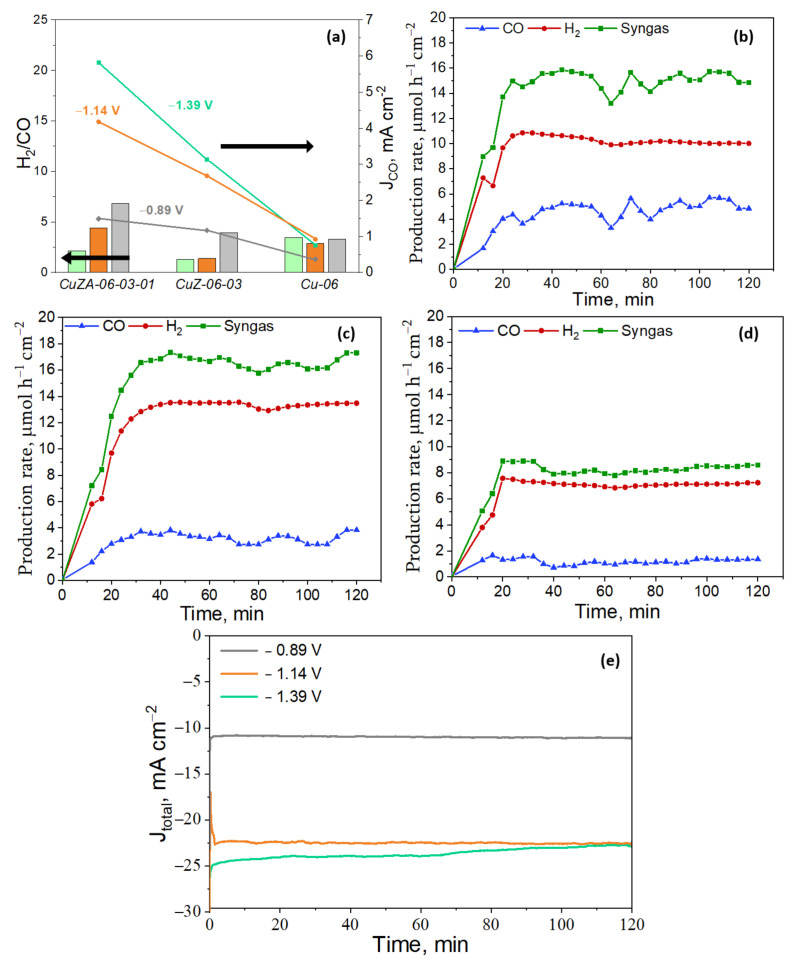
(**a**) H_2_/CO ratio and the corresponding CO current density (J_CO_) at different applied potentials of the CuZnAl-oxide based electrodes, the black arrows indicate that bars belong to the H_2_/CO ratio axis and the lines belong to the Jco axis. Production rates of H_2_ and CO on the CuZA-06-03-01 electrode at different applied potentials: (**b**) −1.39 V; (**c**) −1.14 V; and (**d**) −0.89 V vs. RHE. (**e**) Evolution time of the total current density of the ER-CO_2_ on the CuZA-06-03-01 electrode at different applied potentials.

As shown in Figure 13a, the partial current density for CO formation increased by increasing the applied potential for all of the tested electrodes. This trend was more marked on the CuZA-06-03-01 electrode, reaching 5.82 mA cm^−2^ at −1.39 V vs. RHE, which is comparable to the previously reported Cu-based electrodes performance [60]. Moreover, a tunable H_2_/CO ratio was achieved with this ternary cathode. The H_2_/CO ratio reached ~7 at −0.89 V vs. RHE, and it dropped with more negative applied potentials (~4 at −1.14 V vs. RHE and ~2 at −1.39 V vs. RHE). In the case of the CuZ-06-03 catalyst, the H_2_/CO ratio was almost 1 at the higher applied potentials and ~4 at −0.89 V vs. RHE. On the contrary, the Cu-06 led to almost the same H_2_/CO ratio of 3 at all of the reduction applied potentials. Nowadays, the ER-CO_2_ to syngas with the tunable H_2_/CO ratio is regarded as a promising method for generating numerous energy-dense chemicals by well-established processes. In this context, depending on its composition, syngas can be used to generate different derivatives or fuels. For example, it can be directly utilized to produce methanol (with H_2_/CO ratio of ~2), ethanol (with H_2_/CO ratio of ≤1), and Fischer-Tropsch products (with H_2_/CO ratio of ≥2) [6]. Therefore, the here reported CuZA-06-03-01 catalyst can be potentially exploited in Fischer-Tropsch synthesis processes by applying more negative potentials (H_2_/CO ≥ 2) and the synthesis of methanol at the most positive applied potentials (H_2_/CO~2). Moreover, the CuZ-06-03 can be used in the Fischer-Tropsch synthesis (H_2_/CO ≥ 2 at −0.89 V vs. RHE) and ethanol production (H_2_/CO~1) at more negative potentials. Meanwhile, the Cu-06 catalyst could be used in combination with the Fischer-Tropsch synthesis technologies at different applied potentials.

The production rates of H_2_ and CO were determined by micro GC every 4 min. The production yields of both gases reached a constant value after 20 min of CO_2_ electrolysis on the CuZA-06-03-01 cathode at the different tested potentials, as can be seen in Figure 13b–d. The production rate of syngas at these CuZnAl-oxide based electrodes is one order of magnitude lower than in the other similar studies, which have reached up to 100 µmol h^−1^ cm^−2^ [6,60,61,62]. However, it could be explained by the 5-fold lower catalyst loading used in this work, with respect to the other studies [60].

The stability for the CO_2_ co-electrolysis on the CuZA-06-03-01 electrode has been investigated during 2 h. As shown in Figure 13e, the overtime duration of the total current density profiles is very stable, without any significant current changes at the lower applied potentials, reaching ~24 mA cm^−2^. However, the total current density drops down (10%) at −1.39 V vs. RHE after 60 min of CO_2_ co-electrolysis. This fact could be attributed to the difficulty of managing the transport of gaseous products through the pores of the catalytic layer during high rates of co-electrolysis. The bubble accumulation could block the catalytic sites due to mass-transfer limitations, partially caused by the non-porous glassy carbon, which is the conductive support of the RDE system [63].

**Figure 14:**
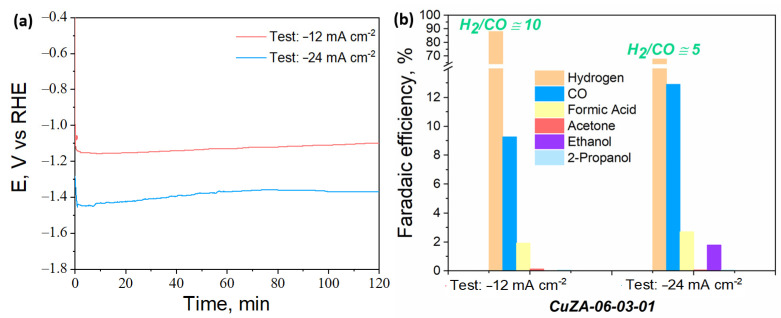
(**a**) Time evolution of the cathodic potential and (**b**) Faradaic efficiencies for the ER-CO_2_ on the CuZA-06-03-01 GDEs tested at −12 and −24 mA cm^−2^.

To reduce the issue of bubbles accumulation on the catalysts surface, the CuZA-06-03-01 catalyst was deposited into a porous and conductive substrate to form a GDE and was tested in an H-cell system in galvanostatic mode by reaching the same current densities obtained in the RDE system. Therefore, two RDE tests were imitated, the ones at −0.89 and −1.39 V vs. RHE, corresponding to a current density of −12 and −24 mA cm^−2^, respectively (see Figure 13e). As shown in Figure 14a, the registered cathodic potentials were stable during the tests, and they were not significantly different from the applied potentials in the RDE system. It is important to mention that a tunable H_2_/CO ratio was also achieved in this case, in the H-type cell setup with the ternary catalyst, as shown in Figure 14b. The H_2_/CO ratio was ~5 at −24 mA cm^−2^, and it was raised to ~10 with the more positive applied current density, as in the RDE setup. Although the trend of syngas vs. applied current density was also verified in the H-type cell setup, the H2/CO ratio values increased with respect to the values obtained in the RDE tests. Based on these results, a possible reason for the high production of hydrogen with respect to CO is proposed. Unlike the H-type cell configuration, the RDE system allows the performance of tests without mass transfer limitations, which improves the overall Faradaic selectivity for CO_2_ reduction reaction with a decreasing rate of HER [56].

The ex-situ FESEM and XRD characterization, shown in Figure 9 and Figure S5 in the SI, evidence that the original particles of the CuZA-06-03-01 electrodes and the crystalline phase composition have changed during the test. The catalyst is transformed into agglomerated spherical nanoparticles during the electrochemical reaction. These nanoparticles become smaller at the highest applied current density, as shown in Figure 9e,g. In addition, the diffraction peaks in the XRD graph of the tested electrode, shown in Figure S5, belong to monoclinic CuO (JCPDS number: 00-048-1548), Cu_2_O (JCPDS 00-005-0667), hexagonal ZnO (JCPDS number: 00-036-1451), and graphite (JCPDS number: 00-041-1487) crystalline phases. The electrode tested at the highest applied current density of −24 mA cm^−2^ (Figure S5a) seems to have less CuO in the bulk than the one tested at −12 mA cm^−2^, while both of them contain Cu_2_O and probably only small amounts of metallic Cu, which are not visible from the XRD spectra. Moreover, a comparable result has been obtained with the XPS analysis applied to fresh and tested electrodes. A clear decrease in the amount of CuO has been detected in the tested electrodes (see Figure S7 and Table S2 in SI) from both the Cu2p HR spectra and from the indirect calculation of the relative abundance of Cu^2+^ and Cu^0^ + Cu^1+^ oxidation states. The sample tested at −24 mA cm^−2^ has shown the lowest amount of Cu^2+^ equal to 66%, compared to 100% of the fresh electrode and 77% of the electrode tested at the lowest current density. The increased CuO reduction to an oxide-derived-Cu (i.e., Cu_2_O) under the highest negative potentials, makes the CuZA-06-03-01 electrode more selective to the CO_2_ conversion products and suppresses the HER, as shown in Figure 14b. It is possible that increasing the Cu_2_O content contributes to the decrease in the CO affinity leading to its facile desorption, and thus gaseous CO production [60]. In addition, the increased ethanol production at −24 mA cm^−2^ suggests the reconstruction of the catalyst towards the formation of specific active sites, e.g., mixed Cu oxidation states such as Cu^0^ and Cu^+1^, which are able to perform the *CO dimerization to more reduced products of the CO_2_ [19,45,64]. This finding is in agreement with the lowest Auger parameter (1848.6 eV) and the highest relative abundance of Cu^0^+Cu^1+^ oxidation states (34%) in this electrode observed from the XPS analysis after the test (see Table S2 in SI). Furthermore, other than the syngas tunability at different applied potentials, these results demonstrate the potential of the addition of ZnO and Al_2_O_3_ in the Cu-based catalyst to stabilize the Cu_2_O species and avoid its complete reduction to Cu^0^ under the CO_2_ electroreduction conditions, which is a strategy that could be employed to tune the formation of more reduced CO_2_ conversion products such as C_2+_.

## 4. Conclusions

Three CuZnAl-oxide based mesoporous electrocatalysts were synthesized by the co-precipitation method, while maintaining the concentration of each metal nitrate used as precursor. Each of the prepared catalysts had only CuO in the bulk and a different Cu^0^ + Cu^1+^ + Cu^2+^ surface composition. It was found that the CuZA-06-03-01 catalyst, which has the lowest amount of Cu^0^ and Cu^1+^ on the surface, was the most prone for catalyzing the ER-CO_2_ to produce syngas. In addition, the ex-situ measurements performed on tested electrodes revealed a superior selectivity towards CO_2_ reduction products and a reduced H_2_/CO ratio at the most negative potentials, which is related to the reduction of CuO to stable Cu-oxide derived species such as Cu_2_O, during the electrochemical reaction. This tricomponent material is constituted by mesoporous corsages of nanopyramidal structures and showed the highest total catalytic activity (90 mA cm^−2^ from LSV measurements) and the best selectivity for CO and H_2_ production (95% at the most positive applied potential) compared to the two other tested electrodes. This catalytic behavior might be related to its lowest diffusional mass transfer resistance, its highest total pore volume, and its low CuO crystal size among all the prepared catalysts. Indeed, by applying different potentials on this ternary catalyst, a tunable H_2_/CO ratio can be obtained. A syngas with a H_2_/CO ratio of ~2 was obtained on the CuZA-06-03-01 tested electrode at the most positive applied potential (−0.89 V vs. RHE), which is a suitable feedstock for further methanol synthesis. The formation of multi-carbon products was lower than 5% at this applied potential. However, the syngas production rate was the highest (~17 µmol h^−1^ cm^−2^) at the most negative applied potential (−1.39 V vs. RHE).

This study highlights the importance of the surface/bulk chemistry of catalysts and the main textural properties in the resulting selectivity towards CO and H_2_ products. The syngas mixture is highly desired since it can be further converted in numerous energy-dense chemicals by well-established processes. In this regard, by systematically tuning the electrocatalyst, it is possible to control the selectivity of the desired reaction. Therefore, by increasing the crystal size (>25 nm) of the CuO, the binding energy of the *CO reaction intermediate could be improved, and so the generation of more reduced chemicals by inducing C-C bonds and C_2+_ products formation. Furthermore, the development of a hierarchical porous structure with an ordered tortuosity may enhance the mass transfer of reactants and products to/from the active catalytic sites, improving the intermediates residence time and promoting the formation of liquid products. Possible strategies could include increasing the calcination temperature or increasing the precursor solution concentration in the synthesis procedure, to pursue the abovementioned changes. On the other hand, these catalysts should be supported onto porous carbon substrates and tested in a different electrochemical system, such as a gas diffusion electrode cell, to enhance mass transport within the cell, reduce ohmic losses, and pursue high current densities (>100 mA cm^−2^) for the achievement of industrially relevant production rates.

## Figures and Tables

**Table 1:** Composition of the prepared ternary catalysts.

Sample	Precursor Concentration, M			Main Composition after Calcination	Electrode Name
	Cu(NO_3_)_2_·3H_2_O	Zn(NO_3_)_2_·6H_2_O	Al(NO_3_)_3_·9 H_2_O		
Cu/Zn/Al	0.6	0.3	0.1	CuO/ZnO/Al_2_O_3_	CuZA-06-03-01
Cu/Zn	0.6	0.3	-	CuO/ZnO	CuZ-06-03
Cu	0.6	-	-	CuO	Cu-06

**Table 2:** Main textural parameters of the synthesized CuZnAl-oxide based catalysts.

Catalyst	BET Surface Area, m^2^ g^−1^	Total Pore Volume, cm³ g^−1^	EDS, Atomic Ratio	Crystallite Size, nm	
				(11-1) Facet of CuO	(100) Facet of ZnO
Cu-06	18.4	0.11	Cu/O 1:1	16.98	-
CuZ-06-03	55.16	0.23	Cu/Zn 60:40	7.08	7.44
CuZA-06-03-01	101.65	0.37	Cu/Zn/Al 60:30:10	8.49	11.40

**Table 3:** Amount of Cu, Zn, and Al by ICP in the investigated samples.

Sample	Molar Composition, % mol		
	Cu	Zn	Al
CuZA-06-03-01	52.3	38.6	9.1
CuZ-06-03	56.9	44.2	-

**Table 4:** Equivalent electrical circuit values.

Electrode	*R_s_*, Ω	*R_t_*, Ω	*C_t_*, µF	*C_dl_*, mF cm^−2^	ECSA, cm^2^
Cu-06	166.1	61.5	3.1	0.2	8.1
CuZ-06-03	160.5	50.5	13.2	4.5	160.2
CuZA-06-03-01	158.9	38.2	4.6	0.6	21.8

## Data Availability

Our study did not involve any data in external sources.

## Supplementary Materials

The following are available online at https://www.mdpi.com/article/10.3390/nano11113052/s1. Figure S1: BJH pore size distribution curves of the prepared CuZnAl-oxide based samples: (a) Cu-06, (b) CuZ-06-03, and (c) CuZA-06-03-01. Figure S2: N_2_ adsorption/desorption isotherms of the prepared CuZnAl-oxide based samples. Figure S3: Additional TEM results for samples Cu-06, CuZ-06-03, and CuZA-06-03-01. From left to right, the analysis consists of a representative Bright-field TEM image of a specific region of the sample, the corresponding electron diffraction pattern, and the average EDS spectrum obtained over the whole region. Figure S4: High-angle annular dark field (HAADF) scanning transmission electron microscopy image (left) with the corresponding EDS elemental maps (right) for Cu, Zn, Al, and O elements in the CuZA-06-03-01 sample. Figure S5: XRD patterns of CuZA-06-03-10 electrodes: Fresh (a), tested at −12 mA cm^−2^ (b) and tested at −24 mA cm^−2^ (c). Figure S6: Cyclic voltammetry responses for the CuZA-06-03-01 electrode in a traditional three-electrode cell in a CO_2_ saturated 0.1 M KHCO_3_ solution at different scan rates. Figure S7: XPS Cu2p HR spectra of CuZA-06-03-10 electrodes: Fresh (black), tested at −12 mA cm^−2^ (red) and tested at −24 mA cm^−2^ (green). Table S1: Analysis of the selected area electron diffraction patterns reported in Figure S3 and in Figure 6 in the manuscript. Table S2: The table shows the percentage of oxidation states of copper and Auger parameter values calculated for CuZA-06-03-10 electrodes and the related average oxidation states.
