# Supplementary material for: CuZnAl-Oxide Nanopyramidal Mesoporous Materials for the Electrocatalytic CO2 Reduction to Syngas: Tuning of H2/CO Ratio
Source: Nanomaterials (Basel). 2021 Nov 13;11(11):3052. doi: 10.3390/nano11113052 (PMC8618478; doi:10.3390/nano11113052)
Supplement: Supplementary file 1 [file nanomaterials-11-03052-s001.zip › nanomaterials-1435171-supplementary.pdf]

Supporting Information

CuZnAl-Oxide Nanopyramidal Mesoporous Materials for the Electrocatalytic CO_2 Reduction to Syngas: Tuning of H_2/CO Ratio

Hilmar Guzmán [1,2], Daniela Roldán [1], Adriano Sacco [2], Micaela Castellino [1], Marco Fontana [2], Nunzio Russo [1] and Simelys Hernández [1,2,*]

[1] CREST Group, Department of Applied Science and Technology (DISAT), Politecnico di Torino,
C.so Duca Degli Abruzzi, 24, 10129 Turin, Italy; hilmar.guzman@polito.it (H.G.); daniela.roldan@polito.it (D.R.); micaela.castellino@polito.it (M.C.); nunzio.russo@polito.it (N.R.)
[2] IIT—Istituto Italiano di Tecnologia, Via Livorno, 60, 10144 Turin, Italy; adriano.sacco@iit.it (A.S.); marco.fontana@iit.it (M.F.)
* Correspondence: simelys.hernandez@polito.it

Summary

S1. BJH pore size distribution of CuZnAl-oxide catalysts 2
S2. Adsorption/desorption isotherms of N_2 at 77 K 4
S3. TEM characterization 5
S4. XRD measurements of the prepared electrodes 8
S5. Freely diffusing or adsorbed on the electrode 9
S6. XPS measurements of the prepared electrodes 10
S7. References 11

S1. BJH pore size distribution of CuZnAl-oxide catalysts

The Figure S1shows that Cu-06 presented a narrow distributions of pore diameters with a mean value of 25 nm; instead the CuZ-06-03 and CuZA-06-03-01catalysts showed a trimodal and bimodal pore size distribution, showing peaks centered at 10 – 23 – 31 nm and at 7 – 15 nm, respectively.

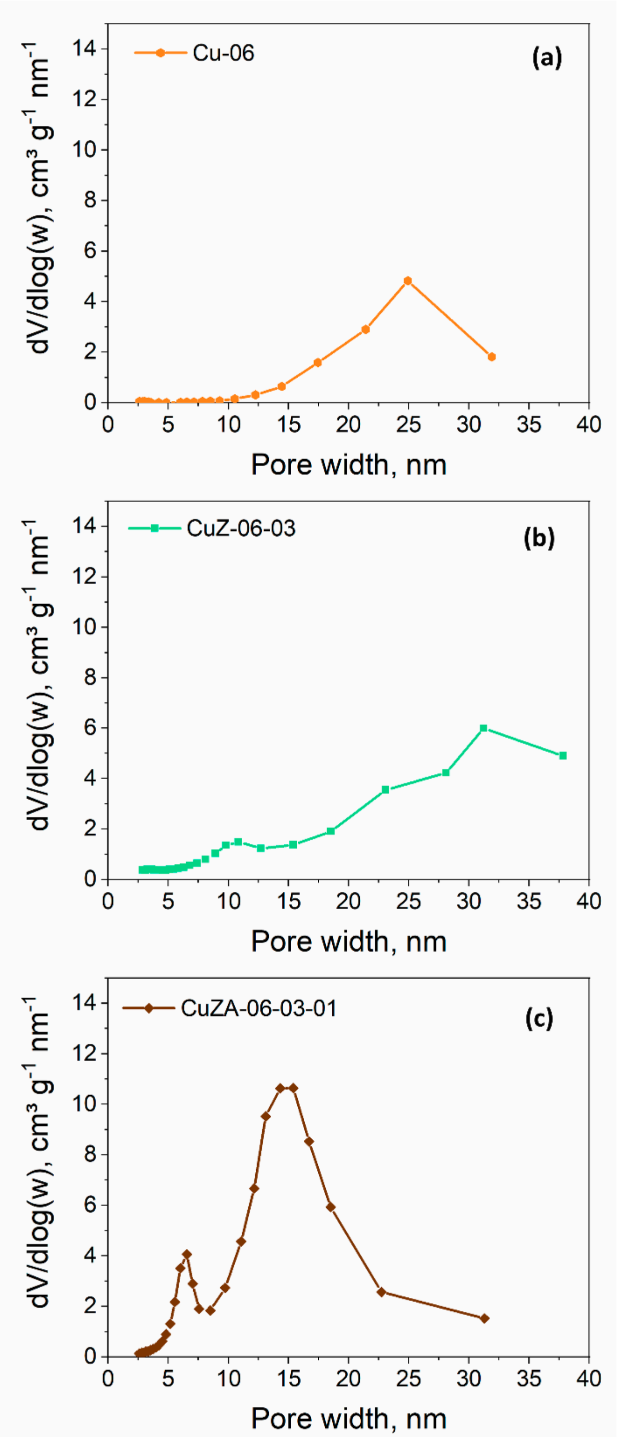

Figure S1. BJH pore size distributions curves of the prepared CuZnAl-oxide based samples: (a) Cu-06, (b) CuZ-06-03, (c) CuZA-06-03-01.

S2. Adsorption/desorption isotherms of N_2 at 77 K

As shown in Figure S2, all isotherms are type III, which are convex in relation to the X axis and do not present monolayer formation. Moreover, the isotherms presented hysteresis loop in the desorption branch. The loops of this type are given by presence of slit-shape mesopores.

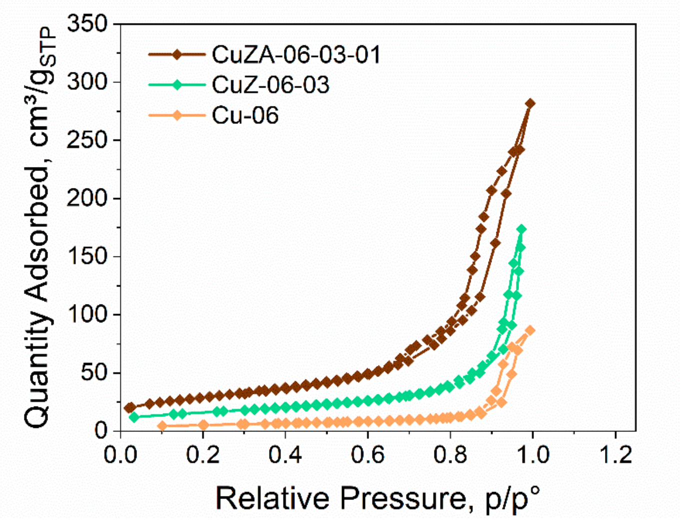

Figure S2. N_2 adsorption/desorption isotherms of the prepared CuZnAl-based oxides samples.

S3. TEM characterization

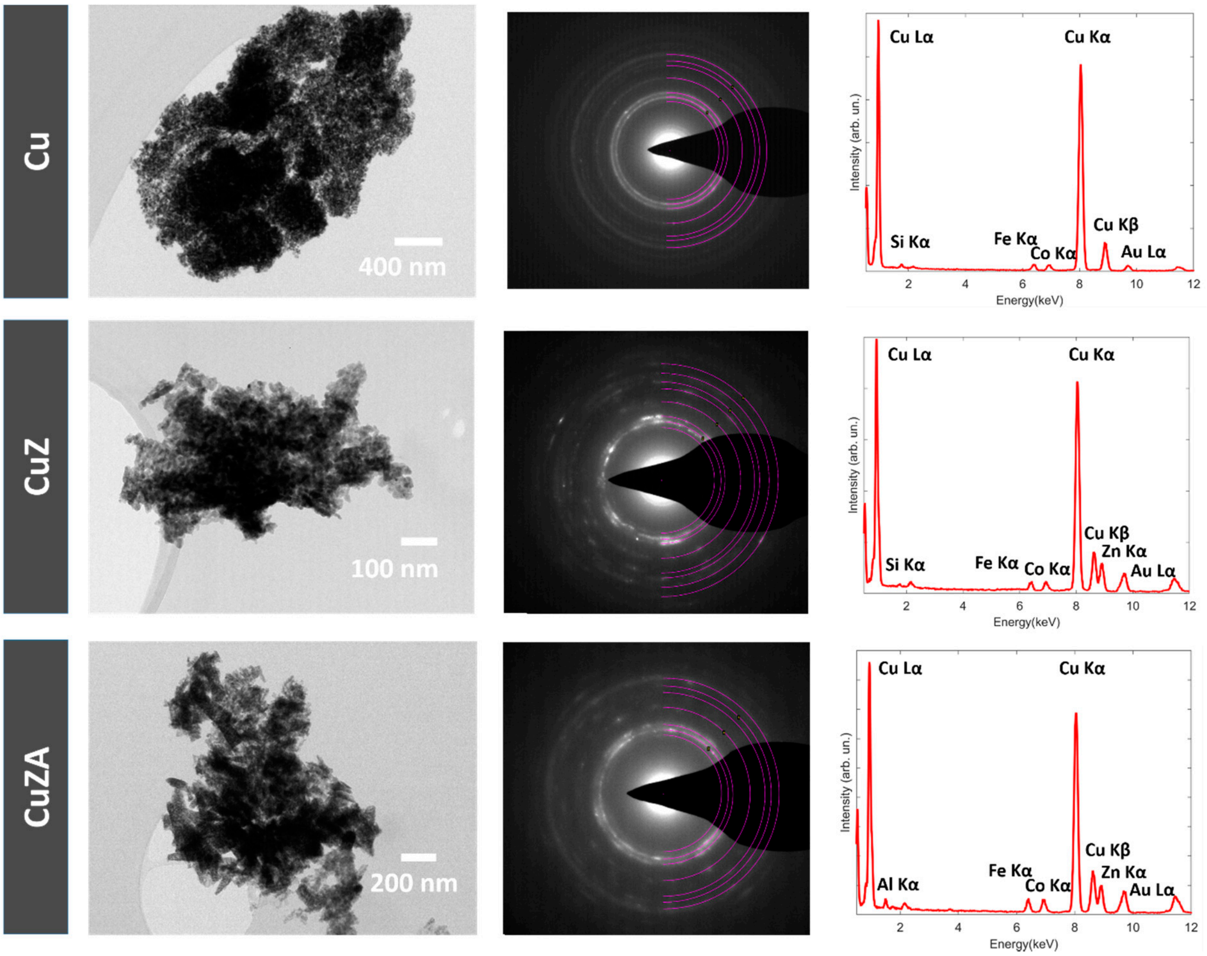

Figure S3. Additional TEM results for samples Cu-06, CuZ-06-03, CuZA-06-03-01. From left to right, the analysis consist of a representative Bright-field TEM image of a specific region of the sample, the corresponding electron diffraction pattern and the average EDS spectrum obtained over the whole region.

Figure S3 reports additional TEM results for all the different samples from specific regions. The analysis of diffraction patterns was carried out by means of the CHT Diffraction analysis script [1] for Digital Micrograph software. The obtained results are reported in Table S1 and they are compared to the reference monoclinic CuO phase (JCPDS number: 00-048-1548, labeled "CuO REF" in the table). Due to the polycrystalline nature of the samples, different families of crystallographic planes manifest themselves as rings in the diffraction pattern; the rings are ordered by increasing radius (expressed as image pixels in the table). It is worth noting that ring 2 and ring 3 are constituted of overlapping contributions from sets of planes with comparable inter-planar spacing. For each sample, the ratio between the inter-planar distance associated to every diffraction ring with respect to the most intense one was calculated; these results are in accordance with the values reported for the monoclinic

CuO phase ("CuO REF"). No detectable signature ring for the ZnO phase is present in the diffraction pattern for the CuZ-06-03 and CuZA-06-03-01 samples. It must be stressed that these results are not in conflict with XRD data, since the ZnO diffraction rings are expected to partially overlap with CuO rings in the context of a SAED pattern. Moreover, the relatively low number of crystals in the analysed electron-transparent regions could also contribute to the low visibility of the ZnO diffraction rings.

Table S1. Analysis of the selected area electron diffraction patterns reported in Figure S3 and in Figure 6 in the manuscript.

CuO REF	(hkl)	d (A)	d/d(111)
	(110)	2.75201	1,18
	(002)	2.53236	1,09
	(11-1)	2.52367	1,08
	(111)	2.32429	1,00
	(200)	2.31315	0,99
	(20-2)	1.86764	0,80
	(202)	1.58227	0,68
	(11-3)	1.50600	0,65

Cu-06	ring	SAED r (px)	d / d(ring3)	assigned (hkl)
	1	409	1.19	(110)
	2	448	1.08	(002)-(11-1)
	3	486	1.00	(111)-(200)
	4	608	0.80	(20-2)
	5	714	0.68	(202)
	6	751	0.65	(11-3)

CuZ-06-03	ring	SAED r (px)	d / d(ring3)	assigned (hkl)
	1	405	1.21	(110)
	2	452	1.08	(002)-(11-1)
	3	489	1.00	(111)-(200)
	4	601	0.81	(20-2)
	5	707	0.69	(202)
	6	752	0.65	(11-3)

CuZA-06-03-01	ring	SAED r (px)	d / d(ring3)	assigned (hkl)
	1	403	1.20	(110)
	2	445	1.08	(002)-(11-1)
	3	482	1.00	(111)-(200)
	4	598	0.81	(20-2)
	5	703	0.69	(202)
	6	746	0.65	(11-3)

However, the presence of nanostructures with the ZnO crystalline phase is confirmed by high-resolution TEM analysis, which is discussed in the manuscript. Moreover, EDS spectra immediately confirm the successful incorporation of the desired elements (Zn, Al) in the CuZ-06-03 and CuZA-06-03-01 samples, as shown in Figure S4, and in accordance with the FESEM analysis. It must be highlighted that Fe and Co peaks in EDS spectra are due to the pole piece of the objective lens inside the TEM, while the Au peaks are generated form the Au grid. Moreover, the internal fluorescence Si peak from the Si(Li) detector is present.

As a further insight into EDS characterization, Figure S4 presents a map analysis of a representative region for the CuZA-06-03-01 sample. The reported results confirm that Zn and Al are incorporated in both nanoparticles and nanopyramids, but with different relative concentrations. In fact, in nanoparticles the concentration of Zn and Al is higher than in nanopyramids, as confirmed by a comparison of the contrast in the EDS maps for the different elements.

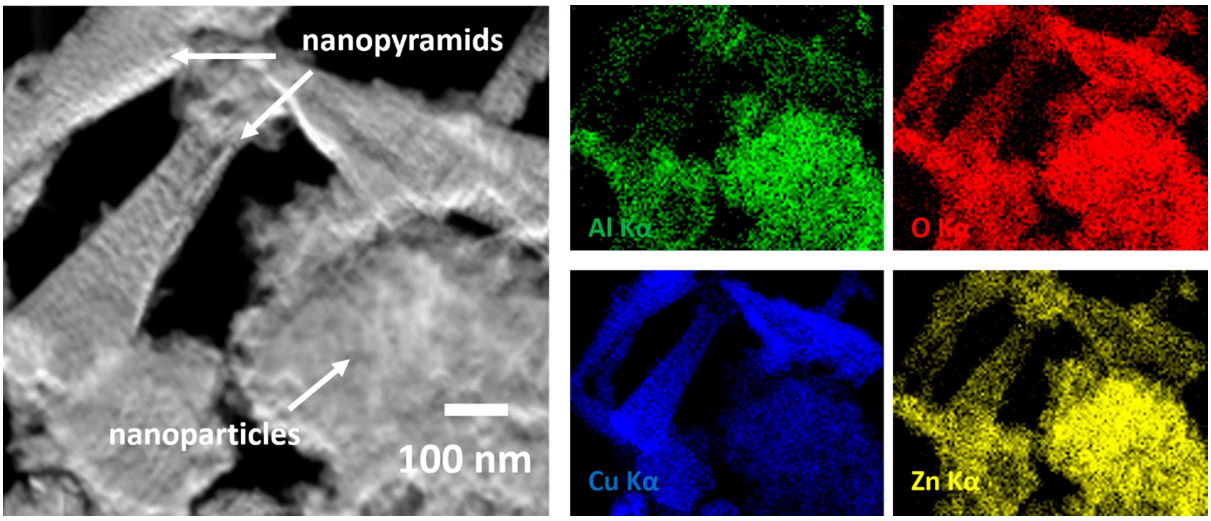

Figure S4. High-angle annular dark field (HAADF) scanning transmission electron microscopy image (left) with corresponding EDS elemental maps (right) for Cu, Zn, Al, O elements in the CuZA-06-03-01 sample.

S4. XRD measurements of the prepared electrodes

A change in the phase composition was observed in the tested electrodes (Figure S5(a) and (b)) with respect to the fresh prepared sample shown in Figure S5(c). In the test carried out at -12 mA cm^{-2}, the (111) facet of CuO disappeared, while the diffraction peaks belonging to Cu_2O (JCPDS 00-005-0667) were identified in the XRD pattern. An increase in the negative applied current density (-24 mA cm^{-2}) resulted in the disappearance of the (11-1) and (202) facets of CuO. Graphite, Teflon and potassium salts residues were also detected due to the carbon substrate, electrolyte and the Nafion used for the tests.

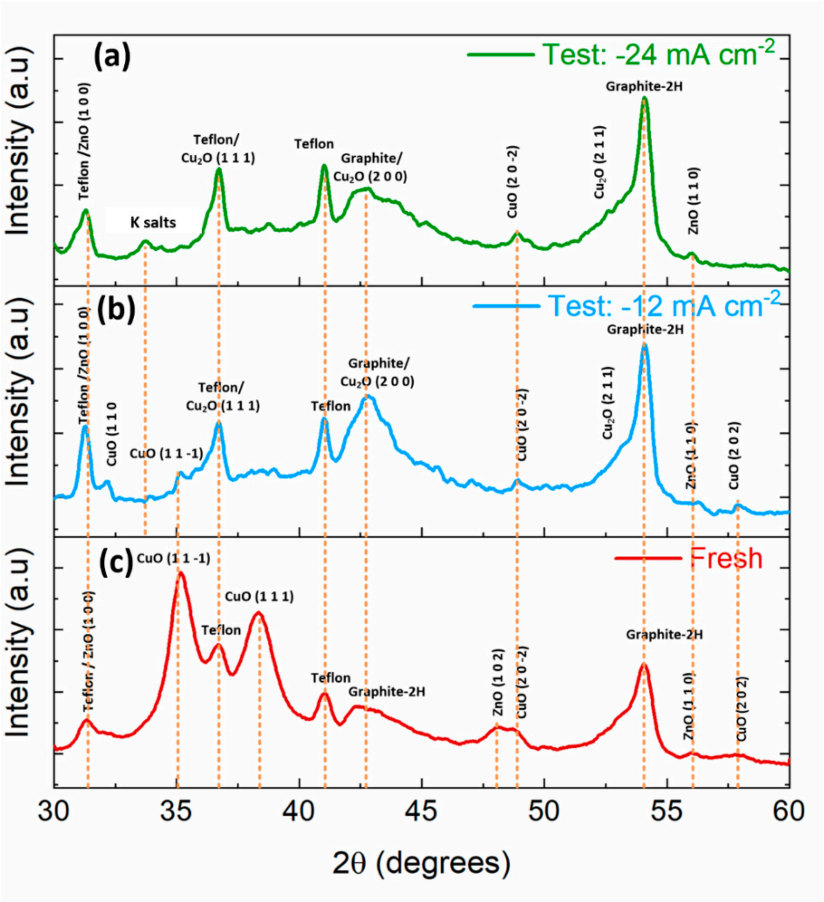

Figure S5. XRD patterns of CuZA-06-03-10 electrodes: fresh (a), tested at -12 mA cm^{-2} (b) and tested at -24 mA cm^{-2} (c).

S5. Freely diffusing or adsorbed on the electrode

The method proposed by Elgrishi, N. et al. was used to determine if the oxidation/reduction peaks observed in the catalysts CVs are prevalently due to freely diffusing molecules or adsorbed species on the electrode surface [2]. Based on this method, for an electrode-adsorbed species, the peak current (i_p) response is expected to vary linearly with the scan rate (v) of the experiment, meaning that a plot of i_p *vs* v should be linear. Unlike, if there is a separation of the peaks with the scan rate, the electrochemical process is quasi reversible, and the analyte is freely diffusing. The results in Figure S6 demonstrate that there are surface-adsorbed species, because the current varies linearly with the scan rate (coefficient of determination R^2 is 0.9943, very close to 1) and no peak to peak separation is observed.

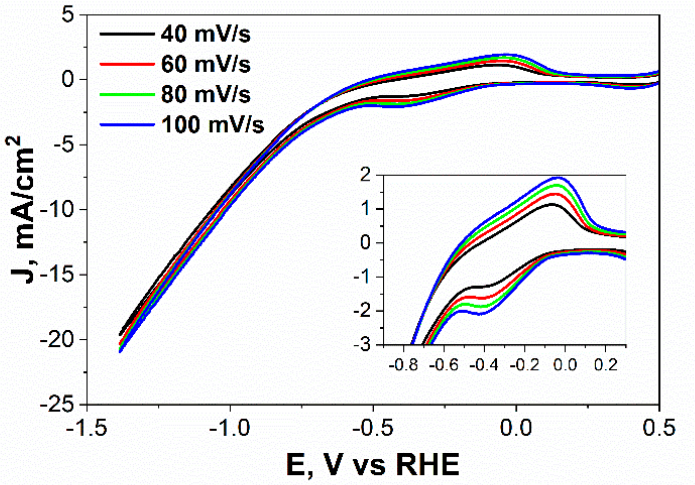

Figure S6. Cyclic voltammetry responses for CuZA-06-03-01 electrode in a traditional 3-electrode cell in CO_2 saturated 0.1 M $KHCO_3$ solution at different scan rates.

S6. XPS measurements of the prepared electrodes

A change in the Cu oxide composition was observed also by means of XPS analysis in the tested electrodes with respect to the fresh prepared sample (see Figure S7). The Cu2p doublet shows a decrease in the Cu^{2+} satellite intensity, due to the increase of the Cu(0) and Cu(1) oxidation states. Also a shift towards lower binding energy, for the tested electrodes, supports our finding related to the change in the oxide composition. As already reported in Figure 7 for powder samples, the relative amount of Cu^{2+} and Cu^{0}+Cu^{1+} have also been calculated (see Table S2), in which a trend is clearly visible: the Cu^{2+} amount passes from 100 % for fresh electrode to the lowest registered amount of 66 % for the tested electrode at -24 mA cm^{-2}. The Auger parameters and their relative correspondent average oxidation states are also in accordance with a change in the Cu oxidation state from +2 to +1.

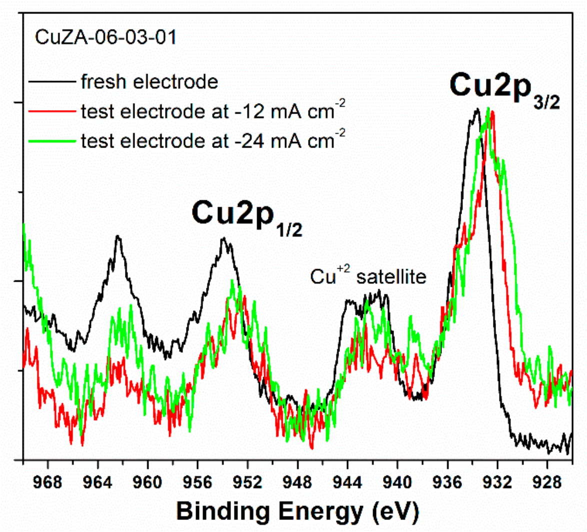

Figure S7. XPS Cu2p HR spectra of CuZA-06-03-10 electrodes: fresh (black), tested at -12 mA cm^{-2} (red) and tested at -24 mA cm^{-2} (green).

Table S2: The table shows the percentage of oxidation states of copper and Auger parameter values calculated for CuZA-06-03-10 electrodes and related average oxidation states.

SAMPLES	Cu^{2+}	Cu^{0}+Cu^{1+}	Auger parameter (eV)	Average oxidation state
Fresh electrode	100	0	1851.4	(II)

Tested at -12 mA cm^{-2}	77	23	1849.2	(I)
Tested at -24 mA cm^{-2}	66	34	1848.6	(I)

S7. References

1 D. R. G. Mitchell, *Ultramicroscopy*, 2008, **108**, 367–374.

2 N. Elgrishi, K. J. Rountree, B. D. McCarthy, E. S. Rountree, T. T. Eisenhart and J. L. Dempsey, *J. Chem. Educ.*, 2018, **95**, 197–206.
